# Efficacy of TACE plus tyrosine kinase inhibitors and immune checkpoint inhibitors in patients with unresectable hepatocellular carcinoma: a systematic review and meta-analysis

**DOI:** 10.3389/fonc.2025.1707622

**Published:** 2025-12-11

**Authors:** Kailin Ye, Jiale Zhou, Jie Lin, Yongzhi Li, Yansong Luo, Fei Xie

**Affiliations:** 1 Department of Hepatobiliary and Pancreatic Surgery, Chengdu Medical College, Chengdu, Sichuan, China; 2 Department of Hepatobiliary and Pancreatic Surgery, The Neijiang First People’s Hospital, Neijiang, Sichuan, China; 3 Department of Hepatobiliary and Pancreatic Surgery, The Second Hospital of Jilin University, Changchun, Jilin, China

**Keywords:** hepatocellular carcinoma, TACE, tyrosine kinase inhibitors, immune checkpoint inhibitors, meta-analysis

## Abstract

**Background:**

Transarterial chemoembolization (TACE) is currently widely used in the treatment of patients with unresectable hepatocellular carcinoma. However, the combination of TACE with tyrosine kinase inhibitors (TKIs) and immune checkpoint inhibitors (ICIs) during treatment remains controversial. This study aims to evaluate the efficacy and safety of TACE combined with TKIs and ICIs in patients with unresectable hepatocellular carcinoma and to compare this approach with other treatment regimens.

**Method:**

We conducted a systematic search of relevant studies from multiple online databases, with the search deadline set at March 2025. We explored the relationship between prognosis and adverse reactions of TACE + TKIs + ICIs combination therapy, TACE + TKIs therapy, and TACE monotherapy in patients with unresectable hepatocellular carcinoma. Based on heterogeneity assessment results, we used fixed- or random-effects models and employed trial sequential analysis (TSA) to determine whether the findings have sufficient conclusive power.

**Result:**

This study included 24 cohort studies involving 3,906 patients. Compared with TACE plus TKIs therapy and TACE monotherapy, the combination therapy of TACE plus TKIs plus ICIs significantly improved the objective response rate (ORR) (risk ratio [RR] = 1.49 [95% confidence interval [CI] = 1.35–1.63], *p* < 0.001 and RR = 1.75 [95% CI 1.48–2.06], *p* < 0.001), disease control rate (DCR) (RR = 1.27 [95% CI 1.20–1.34], *p* < 0.001 and RR = 1.39 [95% CI = 1.23–1.58], *p* < 0.001), and median progression-free survival (mPFS) (mean difference [MD] = 6.05 months [95% CI = 4.77–7.33], *p* < 0.001 and MD = 7.84 months [95% CI = 5.44–10.24], *p* < 0.001), and median overall survival (mOS) (MD = 2.93 months [95% CI = 2.57–3.29], *p* < 0.001 and MD = 4.63 months [95% CI = 2.32–6.95], *p* < 0.001). TSA confirmed that the current sample size was sufficient to support these conclusions. Univariate and multivariate prognostic analyses indicated that the combination therapy of TACE, TKIs, and ICIs, akin to established clinical factors in hepatocellular carcinoma, can serve as a prognostic assessment indicator for patients with unresectable disease. Although the triple therapy carried a slightly higher risk of adverse events compared with the other two treatments, no grade 5 adverse events were observed, indicating that this regimen was generally well tolerated.

**Conclusion:**

This study demonstrates that, compared with TACE combined with TKIs and TACE monotherapy, TACE + TKIs + ICIs combination therapy can substantially improve the prognosis of patients with unresectable hepatocellular carcinoma while maintaining a manageable safety profile.

**Systematic Review Registration:**

https://www.crd.york.ac.uk/prospero/, identifier PROSPERO CRD420250653604.

## Introduction

Liver cancer is the sixth most prevalent cancer worldwide by incidence and the third leading cause of cancer-related mortality, ranking second among men specifically (1). According to GLOBOCAN statistics, in 2022, there were approximately 865,000 new cases of liver cancer and over 750,000 related deaths globally (1). By 2040, it is projected that new liver cancer cases will reach 1.4 million annually, with deaths rising to 1.3 million (2). Hepatocellular carcinoma (HCC) accounts for approximately 90% of all liver cancer cases (3). Due to its insidious onset and rapid progression, most HCC patients are diagnosed at an advanced stage, when surgical resection is no longer an option (4, 5). Retrospective survival analyses indicate that the 3-year survival rate for patients with unresectable hepatocellular carcinoma (uHCC) who did not receive effective treatment is only 17% (6). Given the high prevalence and unfavorable prognosis of advanced hepatocellular carcinoma, there is an urgent need to identify effective treatment strategies to prolong survival and improve the quality of life for patients with uHCC.

Transcatheter arterial chemoembolization (TACE) is the standard first-line treatment for patients with unresectable HCC. It involves embolizing the tumor-supplying arteries and locally releasing chemotherapy drugs to inhibit tumor growth and induce ischemic necrosis (7). However, the efficacy of TACE monotherapy in patients with unresectable hepatocellular carcinoma is often limited by tumor collateral circulation formation, drug resistance, local recurrence, and the risk of distant metastasis (8–10). A meta-analysis of randomized controlled trials showed that, compared with nonactive treatment, TACE can improve the 2-year overall survival rate of patients with unresectable hepatocellular carcinoma, but the benefit is relatively small (11). Tyrosine kinase inhibitors (TKIs) serve as first-line treatments for advanced hepatocellular carcinoma, obstructing tumor angiogenesis by blocking signaling pathways such as vascular endothelial growth factor (VEGF) (12, 13). Relevant randomized controlled trials demonstrate that sorafenib and regorafenib exhibit favorable single-agent efficacy and safety profiles in patients with advanced hepatocellular carcinoma (14–16). Nonetheless, TKIs alone do not achieve the required treatment effectiveness for advanced hepatocellular cancer. A meta-analysis of randomized controlled trials and a multicenter randomized controlled trial revealed that TACE combined with TKIs (TACE + TKIs) produces superior clinical outcomes compared to TACE or TKIs alone (17, 18).

As an innovative cancer therapeutic approach, immune checkpoint inhibitors (ICIs) have shown encouraging clinical efficacy in patients with uHCC (19). ICIs largely enhance antitumor immunity by alleviating immune suppression mediated by Programmed cell death protein 1(PD-1), Programmed death-ligand 1(PD-L1), and Cytotoxic T-lymphocyte-associated protein 4(CTLA-4) (20). Results from global multicenter phase III trials indicate that nivolumab and durvalumab monotherapy demonstrate noninferior overall survival (OS) compared with sorafenib, while the combination of durvalumab and tremelimumab markedly improves overall survival in patients with uHCC (5, 21). Theoretically, combining TACE with TKIs and ICIs (TACE + TKIs + ICIs) can induce ischemia necrosis in tumors, inhibit tumor growth and angiogenesis, augment the release of tumor-specific antigens, and stimulate immune activation to enhance antitumor efficacy (22–25). Ultimately, the combination of TACE, TKIs, and ICIs forms a triple treatment model of “local treatment + systemic control + immune activation”. Currently, extensive multicenter randomized controlled trials are lacking, and debate persists over whether the TACE + TKIs + ICIs combination therapy is more effective than TACE combined with TKIs or TACE monotherapy in patients with uHCC. Therefore, this study aims to evaluate the efficacy and potential adverse effects of TACE + TKIs + ICIs compared with TACE combined with TKIs and TACE monotherapy in the treatment of unresectable HCC by integrating existing clinical research data. Additionally, trial sequential analysis (TSA) is conducted to assess whether the accumulated data have reached the required information size (RIS) and to adjust for increased risks of random error and bias.

## Method

### Search strategy

This study seeks to evaluate the efficacy and safety of TACE combined with TKIs and ICIs in patients with uHCC. The PICO question is, “Does the combination of TACE, TKIs, and ICIs provide superior efficacy and safety compared to TACE combined with TKIs or TACE monotherapy in patients with unresectable hepatocellular carcinoma?” We systematically searched the PubMed, Web of Science, Cochrane Library, Embase, Scopus, OVID, and ProQuest databases for pertinent studies published from the inception of each database up to March 2025. We formulated a search strategy by integrating database-specific medical subject headings (MeSH) with free-text terms. The search keywords included “liver neoplasms”, “chemoembolization, therapeutic”, “TACE”, “tyrosine kinase inhibitors”, “TKI”, “lenvatinib”, “regorafenib”, “apatinib”, “donafenib”, “cabozantinib”, “camrelizumab”, “ramucirumab”, “immune checkpoint inhibitors”, “ICI”, “PD-1”, and “PD-L1”. No language restrictions were enforced throughout the search procedure. To guarantee comprehensive inclusion of relevant literature, the reference lists provided by the relevant studies were also reviewed, with two researchers independently conducting the literature screening.

### Inclusion and exclusion criteria

Studies meeting the following criteria were included in the analysis: (1) Study design: randomized controlled trials (RCTs) or retrospective or prospective cohort studies; (2) Participants: patients diagnosed with HCC based on imaging or pathological biopsy evidence, and those in Barcelona Clinic Liver Cancer (BCLC) stage B/C or with other unresectable HCC; (3) Intervention: TACE combined with TKIs and ICIs; (4) Control measures: TACE combined with TKIs or TACE monotherapy (including conventional TACE and TACE with drug-eluting beads); (5) Outcome measures: studies capable of providing data on median overall survival (mOS), median progression-free survival (mPFS), objective response rate (ORR), disease control rate (DCR), adverse events (AEs), etc.

Studies that did not meet the following criteria were excluded: (1) Systematic reviews, commentaries, case reports, reviews, case–control studies, and literature lacking full text; (2) studies with irrelevant content, noncompliant research methodologies, or inadequate data for extraction or measurement; (3) studies involving patients with BCLC stage D or those who had previously undergone systemic therapy, immunotherapy, or other first-line treatments (including transarterial chemoembolization, hepatic artery infusion chemotherapy, or systemic therapy); and (4) studies published by the same author or utilizing identical data.

### Data extraction and quality assessment

Data extraction was conducted by two independent reviewers using a standardized form. The extracted data comprised study characteristics (first author’s name, year of publication, study design, type of treatment, names of TKIs and ICIs, and sample size); population characteristics (patient age, gender, BCLC stage, Child–Pugh grade, and Eastern Cooperative Oncology Group [ECOG] performance status score [ECOG-PS]); and outcome measures (mPFS, mOS, ORR, DCR, and AEs).

Two researchers independently assessed the quality of the included studies using a standardized quality evaluation instrument. Disputes were resolved through discussion, with a third researcher involved to provide the final decision when necessary. Since all studies in this analysis were cohort studies, the modified Newcastle–Ottawa Scale (NOS) was used to evaluate their quality. Scoring was based on three criteria: selection of the study population, comparability between groups, and evaluation of outcome measures. Each cohort study was assigned a NOS score ranging from 0 to 9 points (7–9 points indicating excellent quality, 4–6 points indicating moderate quality, and 1–3 points indicating low quality).

### Statistical analysis

The included studies were analyzed using Stata 15.0 and Review Manager 5.4. For dichotomous outcome variables, the risk ratio (RR) with a 95% confidence interval (CI) was calculated, and for continuous outcome variables, the mean difference (MD) with a 95% CI was determined. Heterogeneity was evaluated using the Cochrane *Q* test (*p* < 0.1 indicating heterogeneity) and the *I*^2^ test (studies with *I*^2^ values < 25%, 50%, 75%, and 100% were classified as having no, low, moderate, and high heterogeneity, respectively). In cases of low heterogeneity (*I*^2^ ≤ 50% and *p* ≥ 0.1), a fixed-effect model was applied; for moderate to high heterogeneity (*I*^2^ > 50% or *p* < 0.1), sensitivity analyses were performed to identify sources of heterogeneity. If no definitive source was found, a random-effects model was used. Publication bias was evaluated via visual inspection of funnel plots and by Begg’s and Egger’s tests, with *p* < 0.05 considered statistically significant. When publication bias was detected, trim-and-fill analysis was performed.

This study also conducted a TSA to account for the risk of random error. TSA primarily determines the certainty of meta-analysis results by providing the RIS and TSA threshold, similar to the interim monitoring threshold for individual randomized controlled trials (26). In the TSA analysis, the type I error probability (α = 0.05) and type II error probability (β = 0.2) were set, corresponding to a test power of 80%. A relative risk reduction (RRR) of 20% was established for the combination therapy of TACE + TKIs + ICIs compared to TACE + TKIs therapy, and a 30% RRR was set compared to TACE monotherapy for calculating the RIS in binary outcome measures. For continuous outcome measures, the empirical mean difference (MD) and variance were used to calculate the RIS. Depending on the results of the heterogeneity assessment, either a random-effects model or a fixed-effects model was employed.

### Protocol registration

This study is registered in PROSPERO (CRD420250653604).

## Results

### Study selection

**Figure 1:**
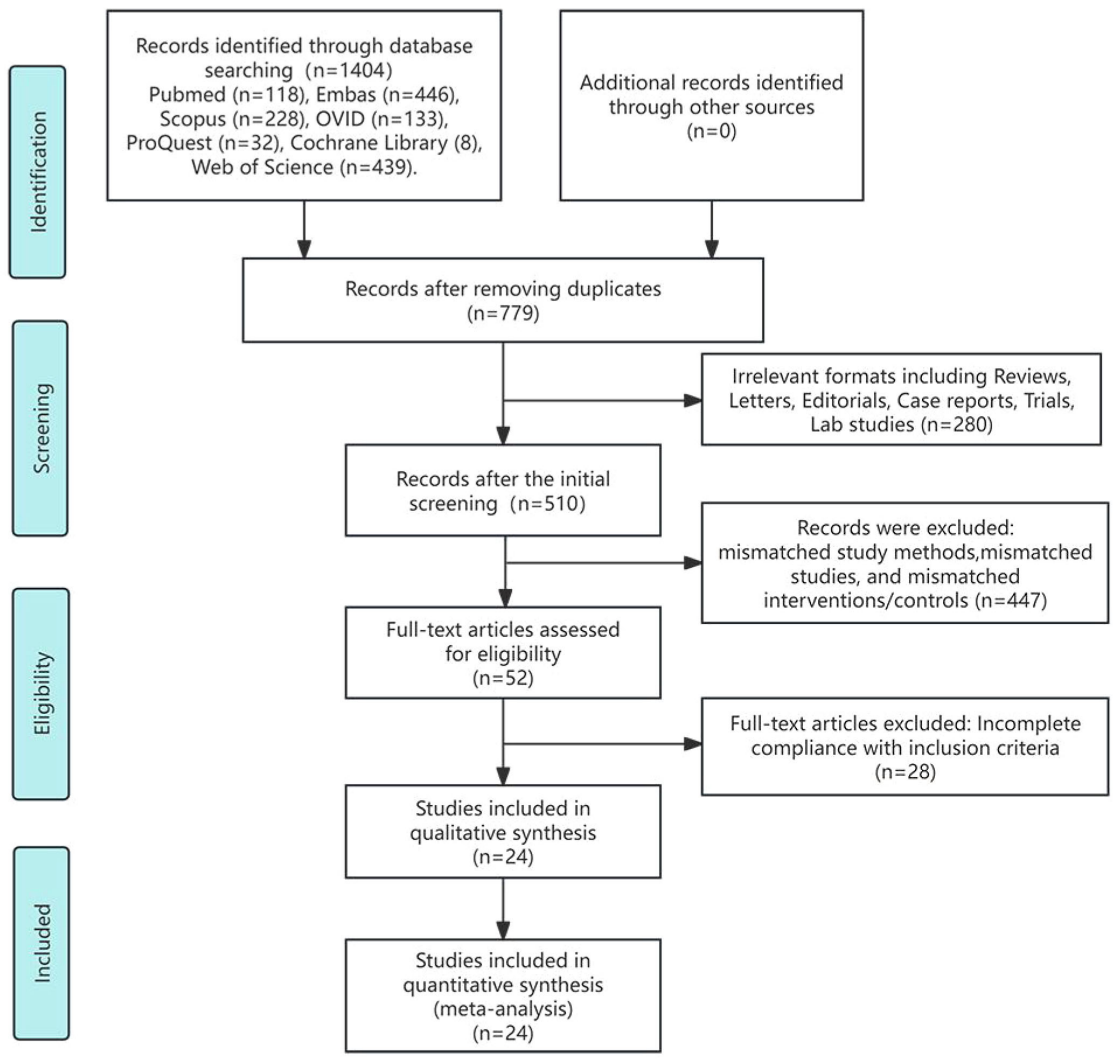
Study inclusion process.

The preliminary database literature search yielded 1,404 study publications. After removing 625 duplicates and 280 studies with irrelevant formats (such as reviews, letters, editorials, case reports, trial protocols, and laboratory research), 510 abstracts were screened. Following abstract screening, 52 studies underwent full-text assessment. During this assessment, we found that the studies by Li et al. and Yuan et al. used the same cohort. We excluded the trial by Yuan et al. because the control measure consisted exclusively of TACE combined with TKIs (27, 28). Ultimately, 24 studies comprising 3,906 participants met the inclusion criteria (28–51). **Figure 1** presents the study inclusion flowchart.

### Study characteristics

Of the 24 studies included, 23 were retrospective cohort studies, and one was a prospective cohort study; eight studies employed propensity score matching (PSM). All studies used TACE in combination with TKIs and ICIs as the intervention. Nineteen studies used TACE combined with TKIs as the control intervention, three studies used TACE combined with TKIs and TACE monotherapy as the control, and two studies used TACE monotherapy as the control. The immune checkpoint inhibitors employed included toripalimab, camrelizumab, sintilimab, tislelizumab, atezolizumab, pembrolizumab, and nivolumab, while the tyrosine kinase inhibitors included sorafenib, lenvatinib, apatinib, regorafenib, and bevacizumab. This meta-analysis comprised 3,906 patients, of whom approximately 82.51% were men, 69.84% had Barcelona Clinic Liver Cancer (BCLC) stage C tumors, 30.11% had a Child–Pugh grade of B, and 46.72% had an ECOG performance status score of 1. The studies and patient characteristics are summarized in **Table 1**. The NOS scores for all included studies were all > 6 points, indicating high-quality literature, as detailed in **Table 2**.

**Table 1:** Baseline characteristics of trial patients included in the meta-analysis.

Study	Research design	Treatment regimen	Number of cases	Male/female	Age (years)	BCLC stage (B/C)	Child–Pugh class (A/B)	ECOG-PS (0/1)
Cai et al. (29)	RCS	TACE + TKIs + ICIs	41	37/4	51.9 ± 10.3	0/41	37/4	33/8
		TACE + TKIs	40	33/7	54.6 ± 11.0	0/40	33/7	28/12
Chen et al. (30)	RCS	TACE + TKIs + ICIs	70	37/33	58.0 (36.0–69.0)	47/23	70/0	27/43
		TACE + TKIs	72	38/34	57.0 (35.0–68.0)	45/27	72/0	30/42
Deng et al. (31)	PSM	TACE + TKIs + ICIs	26	25/1	54.8 ± 11.1	9/17	23/3	8/18
		TACE + TKIs	26	25/1	56.8 ± 9.8	8/18	23/3	4/22
Duan et al. (32)	PSM	TACE + TKIs + ICIs	449	372/77	52.7 ± 8.9	78/371	175/274	262/187
		TACE + TKIs	449	367/82	52.7 ± 9.1	75/374	185/264	256/193
Gao et al. (33)	RCS	TACE + TKIs + ICIs	57	45/12	57.5 ± 9.4	17/40	34/23	48/9
		TACE + TKIs	50	42/8	55.7 ± 11.6	21/29	33/17	40/10
Huang et al. (34)	RCS	TACE + TKIs + ICIs	44	38/6	< 50/≥ 50 (18/26)	9/35	33/11	35/9
		TACE + TKIs	50	45/5	< 50/≥ 50 (17/33)	11/39	38/12	41/9
Li et al. (35)	RCS	TACE + TKIs + ICIs	166	136/30	52.0 (26.0–75.0)	39/127	129/37	79/87
		TACE + TKIs	157	133/24	54.0 (25.0–79.0)	28/129	123/34	66/91
Lu et al. (36)	RCS	TACE + TKIs + ICIs	81	65/16	51.9 ± 12.4	22/59	46/35	0/1/2: 31/35/15
		TACE + TKIs	88	67/21	53.9 ± 12.1	28/60	48/40	0/1/2: 33/41/14
Gao et al. (37)	PSM	TACE + TKIs + ICIs	37	32/5	53.0 (46.0–58.0)	9/28	28/9	–
		TACE	37	31/6	52.0 (48.0–57.0)	11/26	29/8	–
Han et al. (38)	RCS	TACE + TKIs + ICIs	50	41/9	< 60/≥ 60 (25/25)	A + B/C: 33/17	39/11	0–1/2: 30/20
		TACE + TKIs	76	42/34	< 60/≥ 60 (42/34)	A + B/C: 49/27	55/21	0–1/2: 55/21
		TACE	45	38/7	< 60/≥ 60 (26/19)	A + B/C: 30/15	26/19	0–1/2: 28/17
Li et al. (28)	RCS	TACE + TKIs + ICIs	139	121/18	58.0 ± 11.0	99/40	119/20	118/21
		TACE + TKIs	66	61/5	59.0 ± 12.0	48/18	54/12	58/8
		TACE	317	290/27	58.0 ± 12.0	215/102	270/47	259/58
Lin et al. (39)	RCS	TACE + TKIs + ICIs	60	49/11	53.4 ± 11.4	0/60	44/16	23/37
		TACE + TKIs	72	59/13	53.7 ± 11.6	0/72	50/22	18/54
		TACE	82	73/9	54.4 ± 14.1	0/82	62/20	28/54
Qu et al. (45)	PCS	TACE + TKIs + ICIs	56	51/5	51.0 (24.0–82.0)	17/39	53/3	49/7
		TACE	54	49/5	55.0 (29.0–80.0)	18/36	51/3	45/9
Wang et al. (43)	RCS	TACE + TKIs + ICIs	122	68/54	< 60/≥ 60 (65/57)	83/39	81/41	29/93
		TACE + TKIs	52	32/20	< 60/≥ 60 (32/20)	33/19	35/17	17/35
Wang et al. (50)	RCS	TACE + TKIs + ICIs	45	42/3	54.0 (18.0–79.0)	A + B/C: 11/34	30/15	26/19
		TACE + TKIs	20	15/5	62.0 (26.0–75.0)	A + B/C: 5/15	18/2	7/13
Wu et al. (51)	PSM	TACE + TKIs + ICIs	15	14/1	< 60/≥ 60 (7/8)	11/4	15/0	6/9
		TACE + TKIs	15	13/2	< 60/≥ 60 (7/8)	11/4	14/1	3/12
Zhu et al. (40)	PSM	TACE + TKIs + ICIs	34	29/5	< 60/≥ 60 (23/11)	13/21	30/4	19/15
		TACE + TKIs	68	58/10	< 60/≥ 60 (41/27)	26/42	56/12	34/34
Zhao et al. (44)	RCS	TACE + TKIs + ICIs	23	23/0	52.83 ± 7.14	6/17	19/4	12/11
		TACE + TKIs	32	31/1	57.38 ± 9.44	13/19	27/5	18/14
Wu et al. (41)	RCS	TACE + TKIs + ICIs	18	15/3	56.9 ± 8.1	0/18	18/0	7/11
		TACE + TKIs	23	18/5	58.1 ± 9.4	0/23	21/2	7/16
Xia et al. (47)	PSM	TACE + TKIs + ICIs	28	25/3	< 60/≥ 60 (20/8)	0/28	26/2	7/21
		TACE + TKIs	28	26/2	< 60/≥ 60 (19/9)	0/28	26/2	7/21
Yang et al. (49)	PSM	TACE + TKIs + ICIs	29	26/3	53.7 ± 10.2	0/29	21/8	0/1/2: 5/22/2
		TACE + TKIs	29	26/3	51.3 ± 11.2	0/29	20/9	0/1/2: 6/21/2
Zou et al. (48)	RCS	TACE + TKIs + ICIs	70	26/3	53.6 ± 15.1	0/70	46/24	17/53
		TACE + TKIs	90	77/13	52.3 ± 14.8	0/90	61/29	28/62
Wu et al. (42)	RCS	TACE + TKIs + ICIs	38	34/4	56.0 (51.8–63.8)	0/38	28/10	17/21
		TACE + TKIs	52	43/9	54.0 (48.3–62.0)	0/52	43/9	18/34
Xia et al. (46)	PSM	TACE + TKIs + ICIs	59	53/6	≤ 60/>60 (41/18)	0/59	56/3	12/47
		TACE + TKIs	59	54/5	≤ 60/>60 (43/16)	0/59	57/2	12/47

RCS, retrospective cohort study; PCS, prospective cohort study; PSM, propensity score matching; TACE, transarterial chemoembolization; TKIs, tyrosine kinase inhibitors; ICIs, immune checkpoint inhibitors; BCLC, Barcelona Clinic Liver Cancer; ECOG, Eastern Cooperative Oncology Group.

**Table 2:** Methodological quality assessment of cohort studies: the Newcastle–Ottawa scale.

Study	Selection				Comparability	Outcome		
	Representativeness of the exposed cohort	Selection of the nonexposed cohort	Ascertainment of exposure	Demonstration that the outcome of interest was not present at the start of the study	Comparability of cohorts based on the design or analysis controlled for confounders	Assessment of outcome	Sufficient follow-up	Adequacy of follow-up of cohorts
Cai et al. (29)	★	★	★	★	★☆	★	★	★
Chen et al. (30)	★	★	★	★	★☆	★	★	★
Deng et al. (31)	★	★	★	★	★★	★	☆	★
Duan et al. (32)	★	★	★	★	★★	★	★	★
Gao et al. (33)	★	★	★	★	★☆	★	☆	★
Huang et al. (34)	★	★	★	★	★☆	★	★	★
Li et al. (35)	★	★	★	★	★☆	★	★	★
Lu et al. (36)	★	★	★	★	★☆	★	☆	★
Gao et al. (37)	★	★	★	★	★★	★	★	★
Han et al. (38)	★	★	★	★	★☆	★	★	★
Li et al. (28)	★	★	★	★	★☆	★	★	★
Lin et al. (39)	★	★	★	★	★★	★	★	★
Qu et al. (45)	★	★	★	★	★☆	★	★	★
Wang et al. (43)	★	★	★	★	★☆	★	★	★
Wang et al. (50)	★	★	★	★	★☆	★	★	★
Wu et al. (51)	★	★	★	★	★☆	★	★	★
Zhu et al. (40)	★	★	★	★	★★	★	★	★
Zhao et al. (44)	★	★	★	★	★☆	★	☆	★
Wu et al. (41)	★	★	★	★	★☆	★	★	★
Xia et al. (47)	★	★	★	★	★★	★	★	★
Yang et al. (49)	★	★	★	★	★★	★	★	★
Zou et al. (48)	★	★	★	★	★☆	★	★	★
Wu et al. (42)	★	★	★	★	★☆	★	☆	★
Xia et al. (46)	★	★	★	★	★★	★	★	★

“★” a point is given for meeting the corresponding criterion; “☆” no points.

### Objective response rate and disease control rate

**Figure 2:**
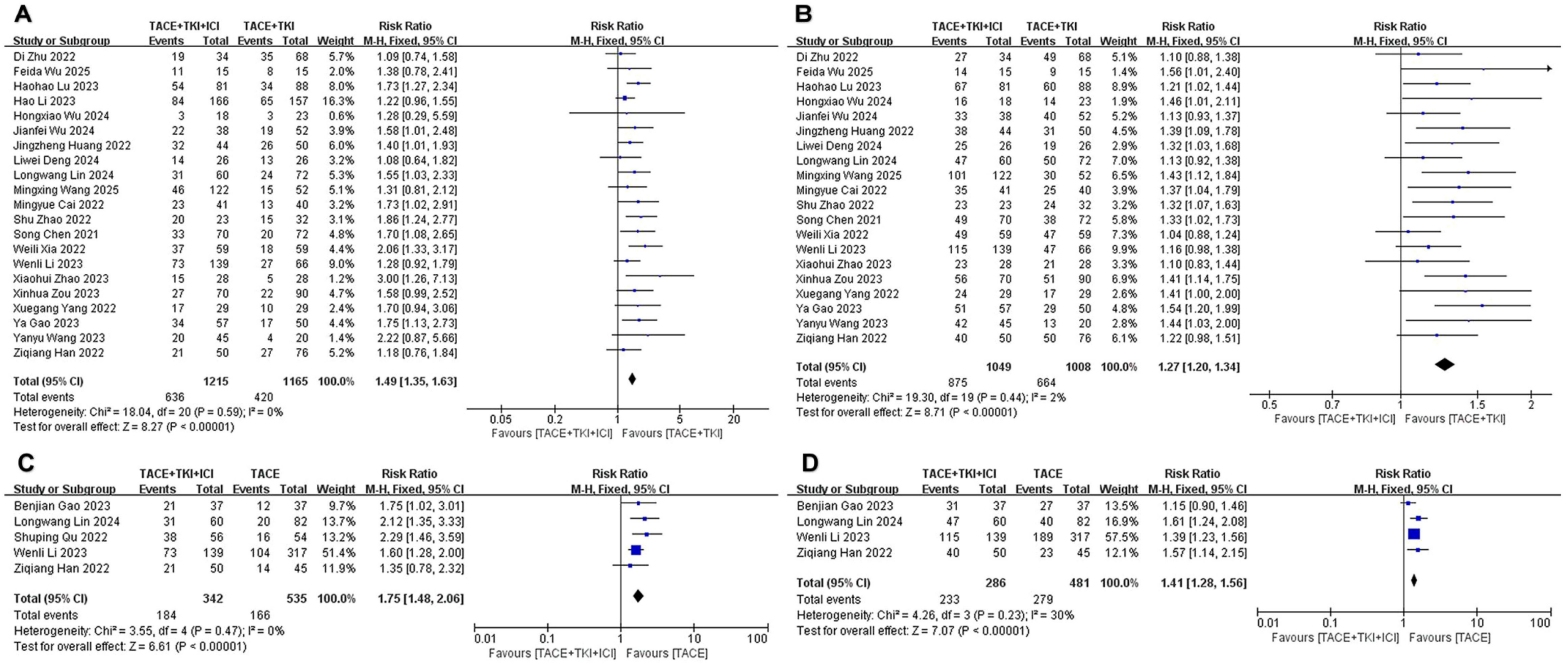
The forest plot depicts the effect of the combination therapy of transarterial chemoembolization (TACE), tyrosine kinase inhibitors (TKIs), and immune checkpoint inhibi-tors (ICIs) on the ob-jective response rate (ORR) and disease control rate (DCR). **(A)** ORR and **(B)** DCR represent comparisons between the TACE + TKIs + ICIs group and the TACE + TKIs group, while **(C)** ORR and **(D)** DCR represent comparisons between the TACE + TKIs + ICIs group and the TACE group. The findings demonstrate that the combined therapy of TACE + TKIs + ICIs significantly enhanced ORR and DCR. Each trial is depicted as a square, with its horizontal position re-flecting the degree of the effect size. The termini of the horizontal bars denote the 95% confi-dence interval (CI), while the diamond signifies the aggregate results of all trials.

Regarding ORR, there was no heterogeneity (*I*^2^ = 15%, *p* = 0.25) among the 22 studies comparing TACE + TKIs + ICIs combination therapy with TACE + TKIs therapy. Subsequent sensitivity analysis was conducted to ensure the correctness and stability of the studies, revealing that the research completed by Duan et al. (32) substantially affected this analysis (**Supplementary Figure S1**). After eliminating this study, the remaining 21 studies exhibited no heterogeneity (*I*^2^ = 0%, *p* = 0.59), and the sensitivity analysis indicated robust stability. A fixed-effect meta-analysis was performed. The results indicated that the ORR in the combination therapy group was significantly higher than that in the TACE + TKIs group (RR = 1.49 [95% CI = 1.35–1.63], *p* < 0.001, **Figure 2A**). The five studies comparing TACE + TKIs + ICIs with TACE monotherapy showed no heterogeneity (*I*^2^ = 0%, *p* = 0.47), and the sensitivity analysis showed satisfactory stability (**Supplementary Figure S1**). A meta-analysis using a fixed-effect model indicated that the ORR in the combination therapy cohort was markedly superior to that in the TACE monotherapy cohort (RR = 1.75 [95% CI = 1.48–2.06], *p* < 0.001, **Figure 2C**).

Regarding DCR, there was moderate heterogeneity (*I*^2^ = 53%, *p* = 0.002) among the 22 studies comparing TACE + TKIs + ICIs combination therapy with TACE + TKIs therapy. Further exploration of the sources of heterogeneity revealed that the studies by Li et al. (35) and Duan et al. (32) had a significant impact on the heterogeneity (**Supplementary Figure S1**). After excluding these two studies, the remaining studies showed no heterogeneity (*I*^2^ = 2%, *p* = 0.44), and a fixed-effect meta-analysis was conducted. The results indicated that the DCR in the combination therapy group was significantly higher than that in the TACE + TKIs therapy group (RR = 1.27 [95% CI = 1.20–1.34], *p* < 0.001, **Figure 2B**). Five studies comparing TACE + TKIs + ICIs vs. TACE monotherapy indicated moderate heterogeneity (*I*^2^ = 67%, *p* = 0.02). Upon examining the origins of heterogeneity, it was shown that the study by Qu et al. (45) significantly influenced heterogeneity (**Supplementary Figure S1**). After excluding this study, the remaining studies showed acceptable heterogeneity (*I*^2^ = 30%, *p* = 0.23), and a fixed-effect meta-analysis was conducted. The results indicated that the DCR in the combination therapy group was significantly higher than that in the TACE monotherapy group (RR = 1.41 [95% CI = 1.28–1.56], *p* < 0.001, **Figure 2D**).

### Median overall survival and median progression-free survival

**Figure 3:**
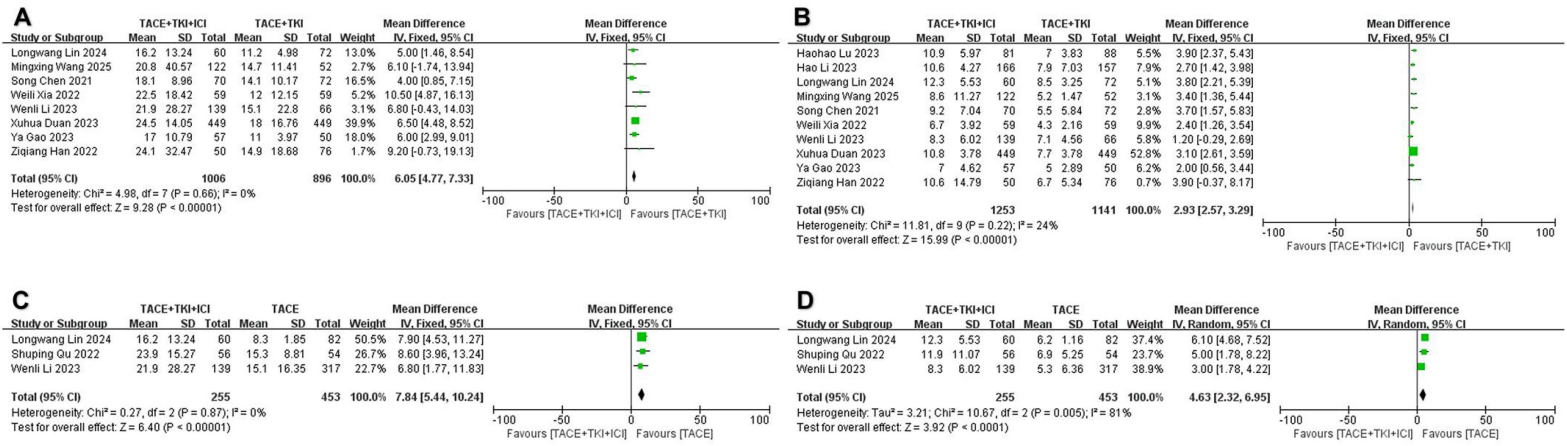
The forest plot illustrates the impact of the combination therapy of transarterial chemoembo-lization (TACE) + tyrosine kinase inhibitors (TKIs) + immune checkpoint inhibitors (ICIs) on median overall survival (mOS) and median progression-free survival (mPFS). **(A)** mOS and **(B)** mPFS represent comparisons between the TACE + TKIs + ICIs group and the TACE + TKIs group, while **(C)** mOS and **(D)** mPFS represent comparisons between the TACE + TKIs + ICIs group and the TACE group. The results indicate that the combination therapy of TACE + TKIs + ICIs significantly improved mOS and mPFS. Each trial is represented by a square, with the po-sition of the square on the horizontal axis indicating the magnitude of the effect size. The ends of the horizontal bars represent the 95% confidence interval (CI), and the diamond rep-resents the overall results of all trials.

All included studies provided data on mOS and mPFS, but to ensure the accuracy of the analysis results, we only combined studies with a sample size of 50 or more in both the experimental and control groups. Regarding mOS, a total of 10 studies met the inclusion criteria when comparing the TACE + TKIs + ICIs combination therapy with TACE + TKIs therapy. A meta-analysis of these 10 studies indicated heterogeneity (*I*^2^ = 48%, *p* = 0.04). To further explore the sources of heterogeneity, we found that the studies by Li et al. (35) and Lu et al. (36) had a significant impact on heterogeneity (**Supplementary Figure S1**). The results demonstrated that the mOS in the combination therapy group was significantly higher than that in the TACE + TKIs therapy group (MD = 6.05 months [95% CI = 4.77–7.33], *p* < 0.001, **Figure 3A**). Three trials satisfied the inclusion criteria when comparing TACE combined therapy with TKIs and ICIs to TACE monotherapy. A meta-analysis of the three investigations revealed no heterogeneity (*I*^2^ = 0%, *p* = 0.87). The sensitivity analysis indicated robust stability, ensuring the accuracy and reliability of the data (**Supplementary Figure S1**). A meta-analysis employing a fixed-effect model indicated that the mOS in the combination therapy cohort was considerably greater than that in the TACE monotherapy cohort (MD = 7.84 months [95% CI = 5.44–10.24], *p* < 0.001, **Figure 3C**).

Regarding mPFS, a total of 10 studies fulfilled the inclusion criteria when comparing TACE + TKIs + ICIs combination therapy with TACE + TKIs therapy. A meta-analysis of these 10 studies indicated no heterogeneity (*I*^2^ = 24%, *p* = 0.22), and the sensitivity analysis demonstrated satisfactory stability (**Supplementary Figure S1**). A meta-analysis using a fixed-effect model showed that the mPFS in the combination therapy group was significantly higher than that in the TACE + TKIs therapy group (MD = 2.93 months [95% CI = 2.57–3.29], *p* < 0.001, **Figure 3B**). When comparing TACE combined therapy with TKIs and ICIs to TACE monotherapy, three studies met the inclusion criteria. Nonetheless, there was high heterogeneity among these three studies (*I*^2^ = 81%, *p* = 0.005). The sensitivity analysis failed to distinctly identify the cause of heterogeneity (**Supplementary Figure S1**). A meta-analysis using a random-effects model showed that the mPFS in the combination therapy group was significantly higher than that in the TACE monotherapy group (MD = 4.63 months, [95% CI = 2.32–6.95], *p* < 0.001, **Figure 3D**).

### Adverse events

No serious adverse events (grade 4 or higher) or treatment-related deaths were documented in any of the studies. Comprehensive information regarding AEs is presented in **Table 3**. No significant difference in the risk of adverse events was observed between the combination therapy of TACE + TKIs + ICIs and TACE + TKIs therapy, with the difference lacking statistical significance (any grade: RR = 1.01 [95% CI = 0.97–1.05], *p* = 0.56; grade 3/4: RR = 1.13 [95% CI = 0.97–1.31], *p* = 0.11. Patients receiving TACE + TKIs + ICIs combination therapy had an increased risk of the following ≥ 3-grade adverse events: hypothyroidism (RR = 5.44 [95% CI = 1.51–19.59], *p* = 0.01), rash (RR = 2.17 [95% CI = 1.27–3.71], *p* = 0.005), pruritus (RR = 3.49 [95% CI = 1.38–8.84], *p* = 0.008), and hyperbilirubinemia (RR = 3.24 [95% CI = 1.14–9.15], *p* = 0.03).

**Table 3:** Summary of treatment-related adverse events.

Adverse events	Any grade				Grades 3 or 4			
	TACE + T + I vs. TACE + T		TACE + T + I vs. TACE		TACE + T + I vs. TACE + T		TACE + T + I vs. TACE	
	RR [95% CI]	*p*-value	RR [95% CI]	*p*-value	RR [95% CI]	*p*-value	RR [95% CI]	*p*-value
Any adverse event	1.01 [0.97–1.05]	0.56	–	–	1.13 [0.97–1.31]	0.11	–	–
Hypertension	1.06 [0.96–1.17]	0.28	12.65 [1.78–90.06]	0.01	1.04 [0.83–1.30]	0.73	6.43 [2.19–18.83]	< 0.001
Diarrhea	1.10 [0.95–1.27]	0.19	5.16 [0.14–193.25]	0.37	1.10 [0.71–1.70]	0.66	8.33 [1.07–65.02]	0.04
Hand–foot syndrome	0.93 [0.84–1.02]	0.12	4.41 [3.16–6.15]	< 0.001	0.96 [0.71–1.30]	0.81	–	–
Hypothyroidism	10.20 [6.11–17.01]	< 0.001	4.66 [1.46–14.86]	0.009	5.44 [1.51–19.59]	0.01	–	–
Rash	1.16 [0.99–1.37]	0.06	3.10 [0.39–24.42]	0.28	2.17 [1.27–3.71]	0.005	–	–
Fever	1.03 [0.95–1.11]	0.51	1.11 [0.93–1.34]	0.24	1.02 [0.71–1.47]	0.91	–	–
Fatigue	1.08 [0.97–1.21]	0.15	2.98 [0.79–11.23]	0.11	1.48 [0.90–2.42]	0.12	3.39 [0.57–20.10]	0.18
Pain	1.00 [0.93–1.08]	0.98	0.91 [0.55–1.49]	0.71	0.95 [0.70–1.29]	0.74	–	–
Thrombocytopaenia	1.03 [0.88–1.20]	0.75	1.27 [1.07–1.50]	0.005	1.17 [0.71–1.91]	0.54	7.13 [1.31–38.78]	0.02
Elevated ALT	1.15 [0.97–1.35]	0.11	1.22 [1.03–1.45]	0.02	1.13 [0.75–1.71]	0.55	0.99 [0.47–2.10]	0.99
Elevated AST	1.15 [0.98–1.36]	0.09	1.12 [0.78–1.61]	0.53	1.45 [0.95–2.21]	0.08	1.32 [0.66–2.65]	0.43
Nausea with or without vomiting	1.07 [0.97–1.19]	0.17	1.34 [1.08–1.67]	0.009	1.24 [0.88–1.74]	0.22	–	–
Decreased appetite	0.97 [0.82–1.14]	0.7	1.12 [0.49–2.59]	0.78	1.24 [0.73–2.08]	0.42	–	–
Decreased WBC	1.19 [0.80–1.75]	0.39	–	–	1.93 [0.51–7.28]	0.33	–	–
Decreased albumin	1.33 [0.87–2.01]	0.18	1.20[0.83–1.73]	0.34	–	–	–	–
Gingival bleeding	1.19 [0.73–1.95]	0.48	–	–	–	–	–	–
Gastrointestinal hemorrhage	0.88 [0.70–1.10]	0.26	1.68 [0.86–3.28]	0.13	0.82 [0.43–1.58]	0.56	–	–
Weight loss	1.42 [1.06–1.91]	0.02	3.55 [2.03–6.21]	< 0.001	–	–	–	–
Dysphonia	0.69 [0.31–1.50]	0.34	–	–	–	–	–	–
Proteinuria	1.04 [0.89–1.22]	0.63	13.90 [8.19–23.59]	< 0.001	0.90 [0.48–1.71]	0.75	–	–
Pruritus	1.71 [1.00–2.93]	0.05	–	–	3.49 [1.38–8.84]	0.008	–	–
Neutropenia	1.51 [0.91–2.51]	0.11	–	–	1.68 [0.37–7.60]	0.5	–	–
Anemia	1.41 [0.82–2.42]	0.22	–	–	–	–	–	–
Lymphopenia	1.69 [0.76–3.76]	0.2	–	–	2.40 [0.30–19.13]	0.41	–	–
Arthralgia	1.52 [0.58–4.01]	0.4	–	–	1.80 [0.33–9.73]	0.5	–	–
Pneumonitis	6.44 [2.08–19.96]	0.001	–	–	8.09 [0.90–72.58]	0.06	–	–
Oral ulcer	1.09 [0.87–1.37]	0.44	–	–	–	–	–	–
Trachyphonia	0.72 [0.33–1.57]	0.4	–	–	–	–	–	–
Hyperbilirubinemia	1.26 [0.93–1.71]	0.14	1.82[0.31–10.59]	0.50	3.24 [1.14–9.15]	0.03	–	–
New ascites	1.22 [0.92–1.62]	0.16	–	–	1.27 [0.56–2.87]	0.56	–	–

RR, risk ratio; ALT, alanine transaminase; AST, aspartate transaminase; TACE, transarterial chemoembolization; T, tyrosine kinase inhibitors; I, immune checkpoint inhibitors.

Compared with TACE monotherapy, patients receiving TACE + TKIs + ICIs combination therapy had an increased risk of the following ≥ 3-grade adverse events: hypertension (RR = 6.43 [95% CI = 2.19–18.83], *p* < 0.001), diarrhea (RR = 8.33 [95% CI = 1.07–65.02], *p* = 0.04), and thrombocytopenia (RR = 7.13 [95% CI = 1.31–38.78], *p* = 0.02).

### Prognostic factor analysis for overall survival and progression-free survival

Exploring independent prognostic risk factors affecting OS and progression-free survival (PFS), multivariate analysis (**Table 4**) revealed that treatment regimen (TACE + TKIs + ICIs/TACE + TKIs/TACE), maximum tumor diameter, Hepatitis B virus (HBV) infection (yes/no), extrahepatic metastasis (present/absent), portal vein tumor thrombus, alpha-fetoprotein level, BCLC stage (C/B), Child–Pugh grade (B/A), and ECOG-PS (1/0) were identified as independent prognostic factors for OS; treatment regimen (TACE + TKIs + ICIs/TACE + TKIs/TACE), maximum tumor diameter, extrahepatic metastasis (present/absent), portal vein tumor thrombus, BCLC stage (C/B), and ECOG-PS (1/0) were identified as independent prognostic factors for PFS. This suggests that the choice of treatment regimen can directly impact the long-term prognosis of patients with unresectable hepatocellular carcinoma.

**Table 4:** Analyses of prognostic factors for survival.

Factor	Overall survival				Progression-free survival			
	Univariate analysis		Multivariate analysis		Univariate analysis		Multivariate analysis	
	HR [95% CI]	*p*-value	HR [95% CI]	*p*-value	HR [95% CI]	*p*-value	HR [95% CI]	*p*-value
Sex								
Female/Male	0.91 [0.80, 1.05]	0.2	–	–	0.90 [0.78, 1.05]	0.19	–	–
Age (years)								
≥ 50/< 50	1.57 [1.01, 2.43]	0.05	–	–	1.24 [0.87, 1.76]	0.23	–	–
≥ 55/< 55	1.25 [0.81, 1.95]	0.31	–	–	1.06 [0.73, 1.55]	0.76	–	–
≥ 60/< 60	1.00 [0.99, 1.01]	0.72	–	–	1.09 [0.93, 1.28]	0.29	–	–
≥ 65/< 65	1.05 [0.72, 1.55]	0.8	–	–	1.11 [0.80, 1.53]	0.54	–	–
ECOG-PS								
1/0	1.23 [1.12, 1.35]	< 0.001	2.06 [1.25, 3.39]	0.004	1.13 [1.01, 1.26]	0.03	1.16 [1.03, 1.32]	0.02
Child–Pugh class								
B/A	1.22 [1.05, 1.43]	0.01	1.95 [1.31, 2.88]	< 0.001	1.07 [0.92, 1.25]	0.39	–	–
HBV infection								
Yes/No	1.28 [1.08, 1.53]	0.005	–	–	1.18 [0.96, 1.44]	0.11	–	–
BCLC stage								
C/B	1.82 [1.28, 2.58]	< 0.001	1.24 [1.02, 1.51]	0.03	–	–	0.91 [0.38, 2.18]	0.84
AFP level (µg/L)								
≥ 200/< 200	1.28 [0.90, 1.81]	0.16	–	–	1.28 [0.72, 2.26]	0.4	–	–
≥ 400/< 400	1.30 [1.16, 1.45]	< 0.001	1.46 [1.14, 1.87]	0.002	1.12 [0.99, 1.27]	0.06	–	–
Tumor distribution								
Multiple/Single	1.16 [0.88, 1.53]	0.28	–	–	1.13 [0.90, 1.43]	0.29	–	–
Hepatic vein invasion								
Yes/No	1.14 [0.96, 1.35]	0.15	–	–	1.21 [0.97, 1.52]	0.09	–	–
Portal vein tumor thrombus								
Yes/No	1.73 [1.46, 2.06]	< 0.001	1.61 [1.15, 2.27]	0.006	1.46 [1.19, 1.78]	< 0.001	–	–
Vp1–2/No	1.16 [0.80, 1.70]	0.43	1.27 [0.68, 2.38]	0.46	1.01 [0.77, 1.33]	0.93	–	–
Vp3–4/No	2.53 [1.54, 4.16]	< 0.001	2.07 [1.21, 3.54]	0.008	1.82 [1.35, 2.46]	< 0.001	1.58 [1.06, 2.37]	0.03
Extrahepatic metastasis								
Yes/No	1.49 [1.33, 1.68]	< 0.001	1.63 [1.35, 1.96]	< 0.001	1.36 [1.18, 1.56]	< 0.001	2.03 [1.52, 2.70]	< 0.001
Largest tumor size								
≥ 5 cm/< 5 cm	1.57 [1.25, 1.97]	< 0.001	2.08 [1.31, 3.30]	0.002	1.23 [0.95, 1.59]	0.11	–	–
≥ 10 cm/< 10 cm	1.72 [1.44, 2.05]	< 0.001	1.34 [1.14, 1.56]	< 0.001	1.45 [1.24, 1.70]	< 0.001	1.35 [1.07, 1.71]	0.01
Treatment option								
TACE + TKIs + ICIs/TACE	0.33 [0.15, 0.75]	0.008	0.30 [0.12, 0.72]	0.007	0.54 [0.38, 0.76]	< 0.001	0.47 [0.33, 0.67]	< 0.001
TACE + TKIs + ICIs/TACE + TKIs	0.46 [0.40, 0.53]	< 0.001	0.42 [0.38, 0.48]	< 0.001	0.49 [0.43, 0.57]	< 0.001	0.41 [0.38, 0.46]	< 0.001

Analyses were performed using the Cox proportional hazard regression model. HR, hazard ratio; CI, conﬁdence interval; ECOG PS, Eastern Cooperative Oncology Group Performance Status; AFP, α-fetoprotein; TACE, transarterial chemoembolization; TKIs, tyrosine kinase inhibitors; ICIs, immune checkpoint inhibitors. HBV, Hepatitis B virus.

### Subgroup analysis of factors associated with patient survival

Subgroup analysis of OS-influencing factors (**Supplementary Figure S2**) showed that, compared with TACE plus TKIs combination therapy, the TACE + TKIs + ICIs triple combination therapy conferred significantly superior survival benefits across all patient subgroups—except for those aged ≥ 60 years, HBV-negative patients, individuals with BCLC stage B disease, and those without hepatic venous tumor thrombus. Similarly, subgroup analysis of PFS-related parameters (**Supplementary Figure S3**) indicated that the TACE + TKIs + ICIs triple combination therapy outperformed TACE plus TKIs combination therapy in all subgroups, with the exception of HBV-negative patients.

### Trial sequential analysis

**Figure 4:**
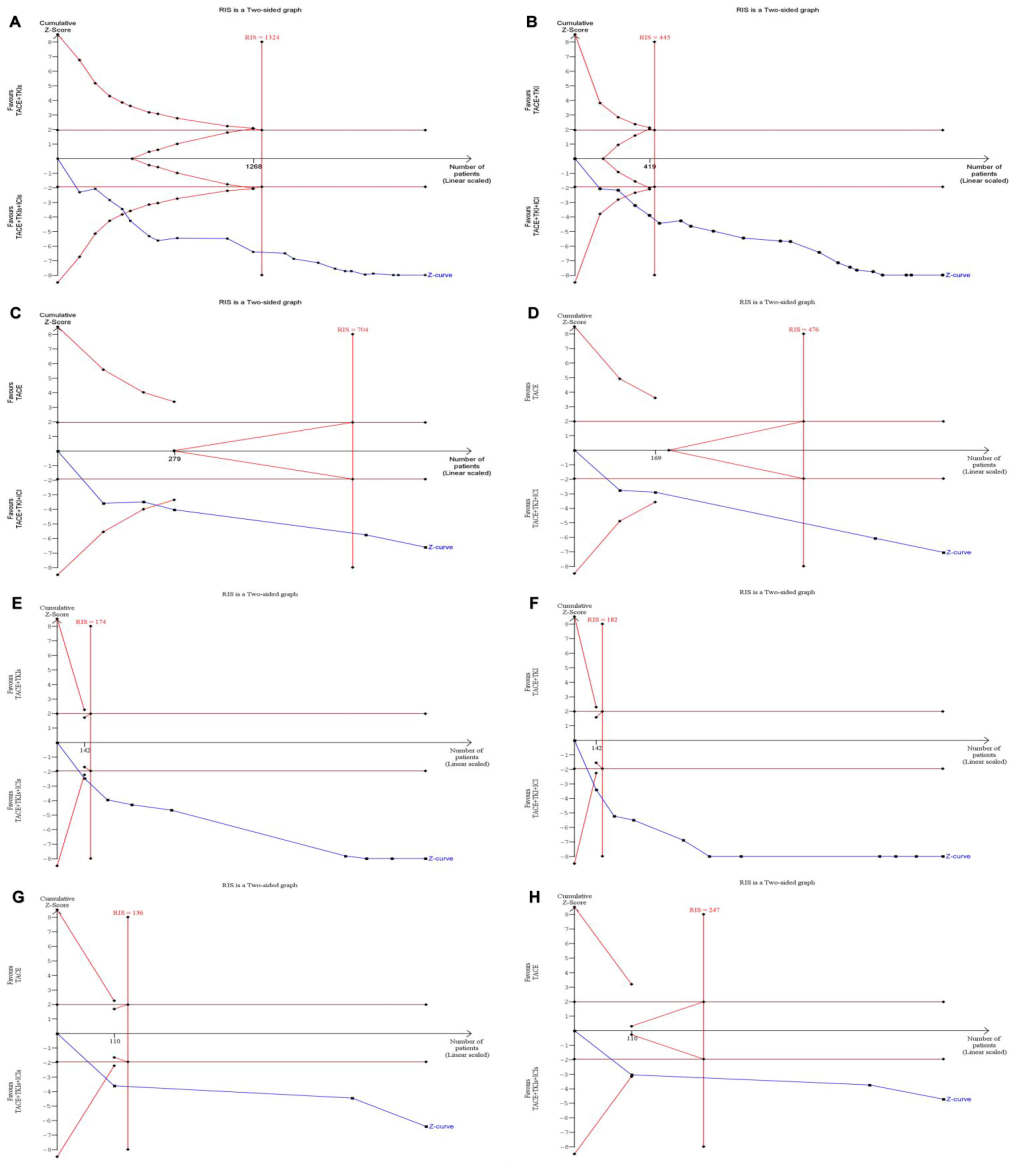
For the sequential analysis of outcome measures between TACE + TKIs + ICIs and TACE + TKIs, a fixed-effect model was used to construct cumulative *Z*-curves, and the expected information volume (RIS) was calculated using the proportion of positive events or the mean difference (MD) and variance in the TACE + TKIs treatment group. **(A)** ORR; **(B)** DCR; **(E)** mOS; **(F)** mPFS. For the sequential analysis of outcome measures between TACE + TKIs + ICIs and TACE, a fixed-effect model or random-effects model was used to construct cumulative *Z*-curves based on heterogeneity, and the expected information content (RIS) was calculated using the positive event rate or empirical MD and variance of the TACE + TKIs treatment group. **(C)** ORR; **(D)** DCR; **(G)** mOS; **(H)** mPFS (analyzed using the random-effects model [SJ]).

A trial sequential analysis of ORR, DCR, mOS, and mPFS was conducted, with results depicted in **Figure 4**. The cumulative *Z*-curves for ORR, DCR, mOS, and mPFS exceeded both the conventional threshold and the TSA threshold, and the cumulative sample size met the expected sample size. The findings demonstrate that this study has recruited an adequate number of patients to derive statistically reliable conclusions and that the combination therapy of TACE + TKIs + ICIs shows statistically significant efficacy differences compared with TACE + TKIs therapy and TACE monotherapy.

### Publication bias

**Figure 5:**
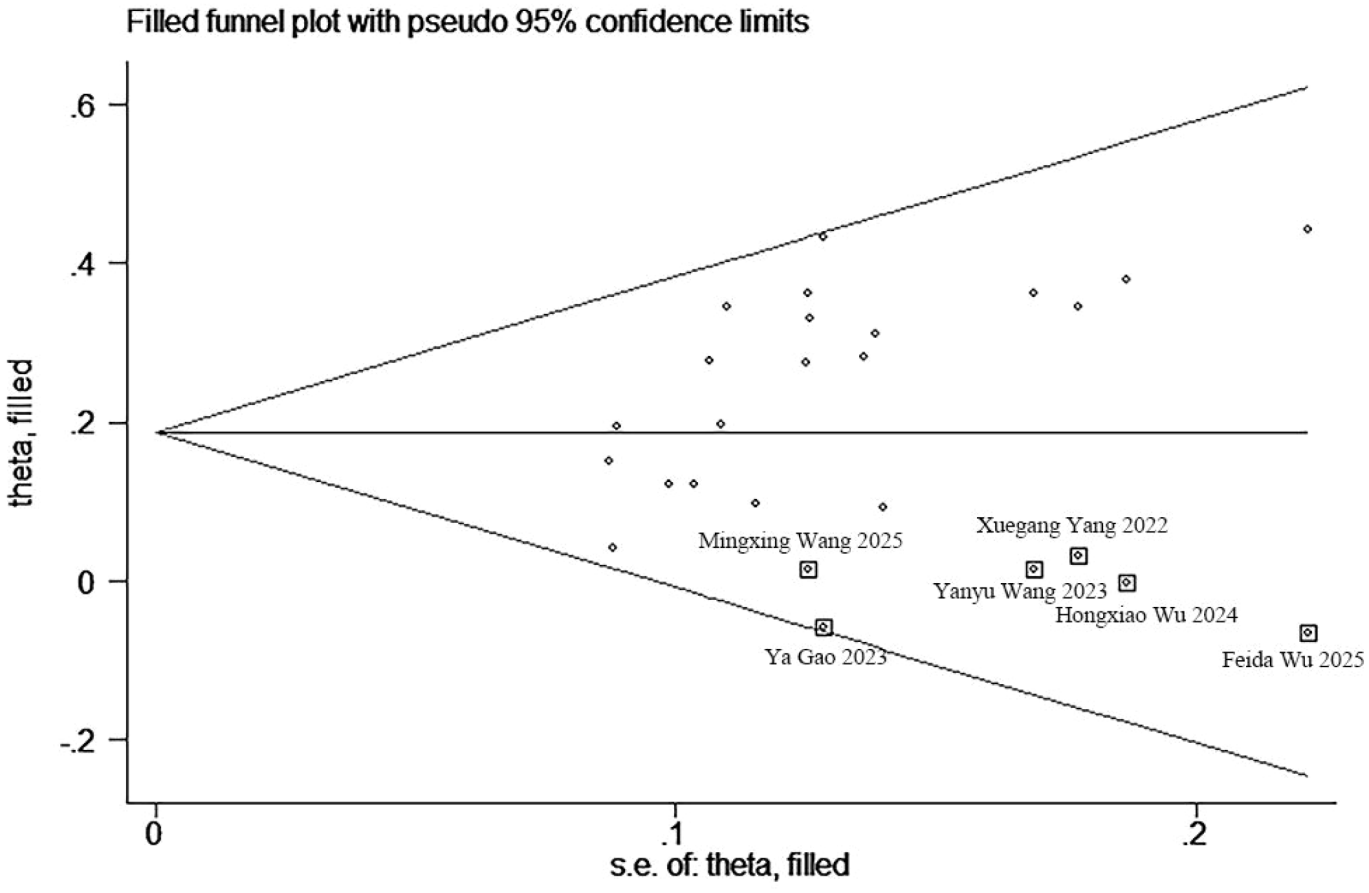
The funnel plot with trim-and-fill for DCR (TACE + TKIs + ICIs vs. TACE + TKIs).

An evaluation of publication bias was conducted, utilizing funnel plots in conjunction with Begg’s and Egger’s tests for ORR, DCR, mOS, and mPFS (**Supplementary Figure S4**). No publication bias was detected between the TACE + TKIs + ICIs combination therapy and TACE + TKIs therapy regarding ORR, mOS, and mPFS, nor between the TACE + TKIs + ICIs combination therapy and TACE monotherapy concerning ORR, DCR, mOS, and mPFS. For DCR between the TACE + TKIs + ICIs combination therapy and TACE + TKIs therapy, the Begg’s test *p*-value was 0.009, and the Egger’s test *p*-value was 0.002, indicating publication bias among the selected 20 studies. Subsequently, the asymmetrical funnel plot was processed using the trim-and-fill method, with the results illustrated in **Figure 5**. The six points within the square indicate the effect sizes of the literature that this study should incorporate in the future. Combined with the funnel plot (**Supplementary Figure S4**), it can be seen that it is necessary to continue including results from six similar studies, including those by Wu et al., Gao et al., Wang et al., and Yang et al. (33, 41, 43, 49–51) to eliminate publication bias.

## Discussion

This meta-analysis assessed the effectiveness and safety of TACE combined with TKIs and ICIs for patients with uHCC. The results indicated that the combination of TACE with TKIs and ICIs considerably enhanced the ORR and DCR, while extending mPFS and mOS, in comparison with TACE plus TKIs therapy and TACE monotherapy. The TSA validated that this study possessed an adequate sample size to derive pertinent findings. This demonstrates that the combination therapy of TACE plus TKIs and ICIs significantly prolongs survival and improves quality of life in patients with uHCC following diagnosis. These findings are consistent with the results reported by Han et al., Gao et al., Lin et al., and Liu et al. (37–39, 52). Combination therapy offers enhanced survival advantages, with the potential mechanisms detailed below. First, TACE induces tumor ischemia and necrosis by embolizing the tumor’s vascular supply, triggering the release of large quantities of tumor-associated antigens. During tumor cell necrosis, metabolic disruption occurs, releasing intracellular lipids, cholesterol, and other substances into the microenvironment. These metabolites act as immunometabolic signals, activating immune effector cells such as T cells and establishing the foundation for immune responses to ICIs. Additionally, the hypoxic and inflammatory milieu following TACE stimulates tumor cells to release large numbers of vesicles bearing PD-L1 on their surfaces. These vesicles circulate throughout the body via the bloodstream, remotely suppressing T-cell function at distant sites. ICIs neutralize PD-L1 on circulating vesicles, thereby reversing vesicle-mediated remote immunosuppression and restoring the body’s antitumor immune response, ultimately reducing the risk of distant tumor metastasis (53–55). Second, TKIs can exacerbate tumor hypoxia and impede tumor angiogenesis while also normalizing the vascular architecture within cancer tissue, thereby facilitating improved infiltration of immune cells into the tumor and producing synergistic antitumor effects with ICIs (24, 56, 57).

After conducting a meta-analysis of all studies included in this review, it was found that treatment regimen, maximum tumor diameter, extrahepatic metastasis, portal vein tumor thrombus, and ECOG-PS were all independent prognostic factors for OS and PFS in uHCC patients, consistent with previous studies (32, 35, 37). Subgroup analysis results indicate that the combination therapy of TACE plus TKIs and ICIs can prolong survival in patients who are HBV-positive, have BCLC stage C disease, and exhibit hepatic venous cancer thrombus. Gao et al. reported that the combination of TACE + TKIs + ICIs resulted in a more significant OS benefit for patients with tumor diameter ≥ 5 cm and BCLC stage C, as well as a superior PFS benefit for patients who are HBV positive, have tumor diameter ≥ 5 cm, and exhibit extrahepatic metastases compared with TACE monotherapy (37). Cai et al. indicated that combination therapy offers enhanced overall survival advantages for patients with more than three tumors or extrahepatic metastases, in contrast to TACE plus TKIs therapy (29). The CHANCE001 research has shown that, compared with TACE monotherapy, triple treatment can prolong PFS and OS for patients with extrahepatic metastases and macroscopically evident portal vein invasion (58). A potential explanation is that TACE therapy efficiently manages intrahepatic lesions but is less efficacious against extrahepatic metastatic lesions, whereas the combination of immunotherapy and targeted therapy can effectively mitigate this therapeutic limitation. Consequently, the combination therapy strategy of TACE, TKIs, and ICIs is essential for uHCC patients, especially those with HBV-positive, BCLC stage C disease, tumor diameter ≥ 5 cm, extrahepatic metastases, and portal vein tumor thrombi, as it offers enhanced survival benefits.

This study demonstrated that the combined therapy of TACE, TKIs, and ICIs was associated with an increased risk of ≥ 3-grade adverse events compared with the other two treatment modalities, including hypothyroidism, rash, pruritus, hyperbilirubinemia, hypertension, diarrhea, and thrombocytopenia. Notably, the findings of this meta-analysis indicated no significant difference in the risk of adverse events of all grades between TACE + TKIs + ICIs therapy and TACE + TKIs therapy. Additionally, none of the included studies reported severe adverse reactions (grade 4 or higher) or treatment-related deaths. A study by CHANCE001 revealed that the occurrence of adverse events in the combination therapy group was marginally elevated compared to the monotherapy group; nevertheless, no grade 5 adverse events were recorded, and the majority of adverse events were easily controlled (58). These findings indicate that, although combination therapy inevitably increases the risk of adverse events relative to other treatments, the severity is predominantly mild to moderate, easily managed or reversible, and within an acceptable range.

The pooled analysis results for mPFS between TACE combined with TKIs and ICIs vs. TACE monotherapy exhibit considerable heterogeneity, attributable to several factors. To ensure the precision of the pooled analysis, we exclusively incorporated studies with sample sizes of 50 or more in both the experimental and control groups, which limited the number of included studies and contributed to heterogeneity. Moreover, although TACE is a conventional therapy for unresectable hepatocellular carcinoma, individualized treatment approaches are frequently used in clinical settings, resulting in considerable variation in drug combinations and treatment protocols across different medical institutions. These differences in TACE regimens likely contributed to the observed heterogeneity.

This meta-analysis has several limitations. First, only one of the included studies was a prospective cohort study; the rest were retrospective cohort studies, which may be susceptible to selection and recall bias. Consequently, extensive, high-quality randomized controlled trials are needed to provide more reliable evidence. Second, owing to the limited availability of data, this study did not perform a subgroup analysis of survival-related factors between the TACE + TKIs + ICIs combination therapy and TACE monotherapy. Finally, because the participants in the analyzed studies were predominantly from Asian populations, the results may not be relevant to Western ones. Additional research, including Western populations, is therefore essential to validate these findings across other racial and geographic groups and to evaluate the effectiveness of this combination therapy in diverse demographics.

## Conclusions

This meta-analysis demonstrates that, compared with TACE plus TKI therapy and TACE monotherapy, the combination therapy of TACE plus TKIs and ICIs provides superior clinical benefits for patients with unresectable hepatocellular carcinoma. It prolongs overall survival and exhibits an acceptable safety profile. We recommend TACE combined with TKIs and ICIs as the preferred treatment option for patients meeting the following criteria: HBV-positive, BCLC stage C, maximum tumor diameter ≥ 5 cm, presence of extrahepatic metastases, and portal vein tumor thrombus. In the future, with the discovery of new immune targets and pathways, such as TIGIT and CD155, combination therapies for hepatocellular carcinoma may achieve further breakthroughs, offering improved clinical treatment benefits (59).

## Data Availability

The original contributions presented in the study are included in the article/**Supplementary Material**. Further inquiries can be directed to the corresponding author.

## References

[1] Bray F Laversanne M Sung H Ferlay J Siegel RL Soerjomataram I . Global cancer statistics 2022: GLOBOCAN estimates of incidence and mortality worldwide for 36 cancers in 185 countries. CA Cancer J Clin. (2024) 74:229–63. doi: 10.3322/caac.21834, PMID: 38572751

[2] Rumgay H Arnold M Ferlay J Lesi O Cabasag CJ Vignat J . Global burden of primary liver cancer in 2020 and predictions to 2040. J Hepatol. (2022) 77:1598–606. doi: 10.1016/j.jhep.2022.08.021, PMID: 36208844 PMC9670241

[3] Llovet JM Kelley RK Villanueva A Singal AG Pikarsky E Roayaie S . Hepatocellular carcinoma. Nat Rev Dis Primers. (2021) 7:6. doi: 10.1038/s41572-020-00240-3, PMID: 33479224 PMC13537433

[4] Forner A Reig M Bruix J . Hepatocellular carcinoma. Lancet. (2018) 391:1301–14. doi: 10.1016/S0140-6736(18)30010-2, PMID: 29307467

[5] Finn RS Qin S Ikeda M Galle PR Ducreux M Kim TY . Atezolizumab plus bevacizumab in unresectable hepatocellular carcinoma. N Engl J Med. (2020) 382:1894–905. doi: 10.1056/NEJMoa1915745, PMID: 32402160

[6] Livraghi T Bolondi L Buscarini L Cottone M Mazziotti A Morabito A . No treatment, resection and ethanol injection in hepatocellular carcinoma: a retrospective analysis of survival in 391 patients with cirrhosis. Italian Cooperative HCC Study Group. J Hepatol. (1995) 22:522–6. doi: 10.1016/0168-8278(95)80445-5, PMID: 7650331

[7] Ikeda M Arai Y Inaba Y Tanaka T Sugawara S Kodama Y . Conventional or drug-eluting beads? Randomized controlled study of chemoembolization for hepatocellular carcinoma: JIVROSG-1302. Liver Cancer. (2022) 11:440–50. doi: 10.1159/000525500, PMID: 36158586 PMC9485929

[8] Li X Feng GS Zheng CS Zhuo CK Liu X . Expression of plasma vascular endothelial growth factor in patients with hepatocellular carcinoma and effect of transcatheter arterial chemoembolization therapy on plasma vascular endothelial growth factor level. World J Gastroenterol. (2004) 10:2878–82. doi: 10.3748/wjg.v10.i19.2878, PMID: 15334691 PMC4572123

[9] Shim JH Park JW Kim JH An M Kong SY Nam BH . Association between increment of serum VEGF level and prognosis after transcatheter arterial chemoembolization in hepatocellular carcinoma patients. Cancer Sci. (2008) 99:2037–44. doi: 10.1111/j.1349-7006.2008.00909.x, PMID: 19016764 PMC11158304

[10] Sergio A Cristofori C Cardin R Pivetta G Ragazzi R Baldan A . Transcatheter arterial chemoembolization (TACE) in hepatocellular carcinoma (HCC): the role of angiogenesis and invasiveness. Am J Gastroenterol. (2008) 103:914–21. doi: 10.1111/j.1572-0241.2007.01712.x, PMID: 18177453

[11] Cammà C Schepis F Orlando A Albanese M Shahied L Trevisani F . Transarterial chemoembolization for unresectable hepatocellular carcinoma: meta-analysis of randomized controlled trials. Radiology. (2002) 224:47–54. doi: 10.1148/radiol.2241011262, PMID: 12091661

[12] Zhou J Sun H Wang Z Cong W Zeng M Zhou W . Guidelines for the diagnosis and treatment of primary liver cancer (2022 edition). Liver Cancer. (2023) 12:405–44. doi: 10.1159/000530495, PMID: 37901768 PMC10601883

[13] European Association for the Study of the Liver . EASL Clinical Practice Guidelines: Management of hepatocellular carcinoma. J Hepatol. (2018) 69:182–236. doi: 10.1016/j.jhep.2018.03.019, PMID: 29628281

[14] Cheng AL Kang YK Chen Z Tsao CJ Qin S Kim JS . Efficacy and safety of sorafenib in patients in the Asia-Pacific region with advanced hepatocellular carcinoma: a phase III randomised, double-blind, placebo-controlled trial. Lancet Oncol. (2009) 10:25–34. doi: 10.1016/S1470-2045(08)70285-7, PMID: 19095497

[15] Bruix J Qin S Merle P Granito A Huang YH Bodoky G . Regorafenib for patients with hepatocellular carcinoma who progressed on sorafenib treatment (RESORCE): a randomised, double-blind, placebo-controlled, phase 3 trial. Lancet. (2017) 389:56–66. doi: 10.1016/S0140-6736(16)32453-9, PMID: 27932229

[16] Abou-Alfa GK Lau G Kudo M Chan SL Kelley RK Furuse J . Tremelimumab plus durvalumab in unresectable hepatocellular carcinoma. NEJM Evid. (2022) 1:EVIDoa2100070. doi: 10.1056/EVIDoa2100070, PMID: 38319892

[17] Li D Pang Y Xu L Xu X . Efficacy and safety of sorafenib combined with TACE in the treatment of advanced hepatocellular carcinoma: A meta-analysis. J BUON. (2021) 26:1355–64. 34564992

[18] Peng Z Fan W Zhu B Wang G Sun J Xiao C . Lenvatinib combined with transarterial chemoembolization as first-line treatment for advanced hepatocellular carcinoma: A phase III, randomized clinical trial (LAUNCH). J Clin Oncol. (2023) 41:117–27. doi: 10.1200/JCO.22.00392, PMID: 35921605

[19] Cheng AL Hsu C Chan SL Choo SP Kudo M . Challenges of combination therapy with immune checkpoint inhibitors for hepatocellular carcinoma. J Hepatol. (2020) 72:307–19. doi: 10.1016/j.jhep.2019.09.025, PMID: 31954494

[20] Narote S Desai SA Patel VP Deshmukh R Raut N Dapsel S . Identification of new immune target and signaling for cancer immunotherapy. Cancer Genet. (2025) 294–295:57–75. doi: 10.1016/j.cancergen.2025.03.004, PMID: 40154216

[21] Yau T Park JW Finn RS Cheng AL Mathurin P Edeline J . Nivolumab versus sorafenib in advanced hepatocellular carcinoma (CheckMate 459): a randomised, multicentre, open-label, phase3 trial. Lancet Oncol. (2022) 23:77–90. doi: 10.1016/S1470-2045(21)00604-5, PMID: 34914889

[22] Huinen ZR Huijbers EJM van Beijnum JR Nowak-Sliwinska P Griffioen AW . Anti-angiogenic agents - overcoming tumour endothelial cell anergy and improving immunotherapy outcomes. Nat Rev Clin Oncol. (2021) 18:527–40. doi: 10.1038/s41571-021-00496-y, PMID: 33833434

[23] Cheu JW Wong CC . Mechanistic rationales guiding combination hepatocellular carcinoma therapies involving immune checkpoint inhibitors. Hepatology. (2021) 74:2264–76. doi: 10.1002/hep.31840, PMID: 33811765

[24] Fukumura D Kloepper J Amoozgar Z Duda DG Jain RK . Enhancing cancer immunotherapy using antiangiogenics: opportunities and challenges. Nat Rev Clin Oncol. (2018) 15:325–40. doi: 10.1038/nrclinonc.2018.29, PMID: 29508855 PMC5921900

[25] Sangro B Chan SL Meyer T Reig M El-Khoueiry A Galle PR . Diagnosis and management of toxicities of immune checkpoint inhibitors in hepatocellular carcinoma. J Hepatol. (2020) 72:320–41. doi: 10.1016/j.jhep.2019.10.021, PMID: 31954495 PMC7779342

[26] Wetterslev J Thorlund K Brok J Gluud C . Trial sequential analysis may establish when firm evidence is reached in cumulative meta-analysis. J Clin Epidemiol. (2008) 61:64–75. doi: 10.1016/j.jclinepi.2007.03.013, PMID: 18083463

[27] Yuan G Li W Zang M Li R Li Q Hu X . Transarterial chemoembolization with/without immune checkpoint inhibitors plus tyrosine kinase inhibitors for unresectable hepatocellular carcinoma: a single center, propensity score matching real-world study. Discov Oncol. (2024) 15:68. doi: 10.1007/s12672-024-00917-1, PMID: 38460053 PMC10924872

[28] Li WL Du YF Yuan GS Zang MG Zhu PL Li R . Analysis of the therapeutic efficacy of transcatheter arterial chemoembolization combined with systemic treatment in unresectable hepatocellular carcinoma. Chin J Clin Oncol. (2023) 50:1135–41. doi: 10.12354/j.issn.1000-8179.2023.20231001

[29] Cai M Huang W Huang J Shi W Guo Y Liang L . Transarterial chemoembolization combined with lenvatinib plus PD-1 inhibitor for advanced hepatocellular carcinoma: A retrospective cohort study. Front Immunol. (2022) 13:848387. doi: 10.3389/fimmu.2022.848387, PMID: 35300325 PMC8921060

[30] Chen S Wu Z Shi F Mai Q Wang L Wang F . Lenvatinib plus TACE with or without pembrolizumab for the treatment of initially unresectable hepatocellular carcinoma harbouring PD-L1 expression: a retrospective study. J Cancer Res Clin Oncol. (2022) 148:2115–25. doi: 10.1007/s00432-021-03767-4, PMID: 34453221 PMC9293824

[31] Deng L Sun Y Wang H Liao C Li D Xu G . Efficacy and safety of transarterial chemoembolization plus donafenib with or without immune checkpoint inhibitors as the first-line treatment for unresectable hepatocellular carcinoma: A propensity score matching analysis. J Hepatocell Carcinoma. (2024) 11:29–38. doi: 10.2147/JHC.S443779, PMID: 38223554 PMC10787561

[32] Duan X Li H Kuang D Chen P Zhang K Li Y . Transcatheter arterial chemoembolization plus apatinib with or without camrelizumab for unresectable hepatocellular carcinoma: a multicenter retrospective cohort study. Hepatol Int. (2023) 17:915–26. doi: 10.1007/s12072-023-10519-8, PMID: 37012542 PMC10386927

[33] Gao Y Lu H Xiong Z . Efficacy and safety of tyrosine kinase inhibitors plus PD-1 inhibitor in patients with transarterial chemoembolization- refractory hepatocellular carcinoma: a two-center retrospective study. Front Oncol. (2023) 13:1231359. doi: 10.3389/fonc.2023.1231359, PMID: 38074659 PMC10702950

[34] Huang JZ Cai MY Huang WS Guo YJ Huang JJ Zhou QF . Transarterial chemoembolization combined with lenvatinib plus programmed death 1 inhibitor for the treatment of unresectable intermediate−advanced hepatocellular carcinoma. Chin J Radiol. (2022) 56:879–85. doi: 10.3760/cma.j.cn112149-20211104-00977

[35] Li H Wang J Zhang G Kuang D Li Y He X . Transarterial chemoembolization combined donafenib with/without PD-1 for unresectable HCC in a multicenter retrospective study. Front Immunol. (2023) 14:1277329. doi: 10.3389/fimmu.2023.1277329, PMID: 38090566 PMC10711098

[36] Lu H Liang B Xia X Zheng C . Efficacy and safety analysis of TACE + Donafenib + Toripalimab versus TACE + Sorafenib in the treatment of unresectable hepatocellular carcinoma: a retrospective study. BMC Cancer. (2023) 23:1033. doi: 10.1186/s12885-023-11535-5, PMID: 37880661 PMC10599044

[37] Gao B Yang F Zheng D Hu S Liu J Liu H . Transarterial chemoembolization combined with tyrosine kinase inhibitors plus immune checkpoint inhibitors for advanced hepatocellular carcinoma: A propensity score matching analysis. J Hepatocell Carcinoma. (2023) 10:2265–76. doi: 10.2147/JHC.S443041, PMID: 38107541 PMC10725696

[38] Han Z Yang F Zhang Y Wang J Ni Q Zhu H . Prognostic efficacy and prognostic factors of TACE plus TKI with ICIs for the treatment of unresectable hepatocellular carcinoma: A retrospective study. Front Oncol. (2022) 12:1029951. doi: 10.3389/fonc.2022.1029951, PMID: 36591442 PMC9798199

[39] Lin LW Nian YX Lin X Ke K Yang WZ Lin JQ . Efficacy and safety of transarterial chemoembolization combined with lenvatinib plus programmed death-1 inhibitor for hepatocellular carcinoma with the hepatic vein and/or inferior vena cava tumor thrombus. Cardiovasc Intervent Radiol. (2025) 48:314–26. doi: 10.1007/s00270-024-03919-2, PMID: 39658748

[40] Zhu D Ma K Yang W Zhou HF Shi Q Ren JW . Transarterial chemoembolization plus apatinib with or without camrelizumab for unresected hepatocellular carcinoma: A two-center propensity score matching study. Front Oncol. (2022) 12:1057560. doi: 10.3389/fonc.2022.1057560, PMID: 36439471 PMC9685301

[41] Wu HX Ding XY Xu YW Yu MH Li XM Deng N . Transcatheter arterial chemoembolization combined with PD-1 inhibitors and Lenvatinib for hepatocellular carcinoma with portal vein tumor thrombus. World J Gastroenterol. (2024) 30:843–54. doi: 10.3748/wjg.v30.i8.843, PMID: 38516240 PMC10950640

[42] Wu J Bai X Yu G Zhang Q Tian X Wang Y . Efficacy and safety of apatinib plus immune checkpoint inhibitors and transarterial chemoembolization for the treatment of advanced hepatocellular carcinoma. J Cancer Res Clin Oncol. (2024) 150:340. doi: 10.1007/s00432-024-05854-8, PMID: 38976071 PMC11230948

[43] Wang MX Lai T Liu AX Wu GY Sun QM Zhang BR . Comparative efficacy of transarterial chemoembolization with and without PD-1 inhibitor in the treatment of unresectable liver cancer and construction and validation of prognostic models. Transl Cancer Res. (2025) 14:383–403. doi: 10.21037/tcr-24-1521, PMID: 39974405 PMC11833367

[44] Zhao S Zhou M Wang P Yang J Zhang D Yin F . Sorafenib, lenvatinib, or lenvatinib combining PD-1 inhibitors plus TACE in unresectable hepatocellular carcinoma: A retrospective analysis. Technol Cancer Res Treat. (2022) 21:15330338221133640. doi: 10.1177/15330338221133640, PMID: 36259214 PMC9583225

[45] Qu S Zhang X Wu Y Men Y Pan H Fang Q . Efficacy and safety of TACE combined with lenvatinib plus PD-1 inhibitors compared with TACE alone for unresectable hepatocellular carcinoma patients: A prospective cohort study. Front Oncol. (2022) 12:874473. doi: 10.3389/fonc.2022.874473, PMID: 35530353 PMC9068979

[46] Xia WL Zhao XH Guo Y Cao GS Wu G Fan WJ . Transarterial chemoembolization combined with apatinib with or without PD-1 inhibitors in BCLC stage C hepatocellular carcinoma: A multicenter retrospective study. Front Oncol. (2022) 12:961394. doi: 10.3389/fonc.2022.961394, PMID: 36249011 PMC9562990

[47] Xia WL Zhao XH Guo Y Hu HT Cao GS Li Z . Transarterial chemoembolization combined with apatinib plus PD-1 inhibitors for hepatocellular carcinoma with portal vein tumor thrombus: A multicenter retrospective study. Clin Transl Gastroenterol. (2023) 14:e00581. doi: 10.14309/ctg.0000000000000581, PMID: 36920551 PMC10208716

[48] Zou X Xu Q You R Yin G . Correlation and efficacy of TACE combined with lenvatinib plus PD-1 inhibitor in the treatment of hepatocellular carcinoma with portal vein tumor thrombus based on immunological features. Cancer Med. (2023) 12:11315–33. doi: 10.1002/cam4.5841, PMID: 36951443 PMC10242346

[49] Yang XG Sun YY Wang HQ Li DS Xu GH Huang XQ . Efficacy and safety of transarterial chemoembolization combining sorafenib with or without immune checkpoint inhibitors in previously treated patients with advanced hepatocellular carcinoma: A propensity score matching analysis. Front Oncol. (2022) 12:914385. doi: 10.3389/fonc.2022.914385, PMID: 36176392 PMC9513034

[50] Wang YY Yang X Wang YC Long JY Sun HS Li YR . Clinical outcomes of lenvatinib plus transarterialchemoembolization with or without programmed death receptor-1 inhibitors in unresectable h-epatocellular carcinoma. World J Gastroenterol. (2023) 29:1614–26. doi: 10.3748/wjg.v29.i10.1614, PMID: 36970591 PMC10037246

[51] Wu FD Zhou HF Yang W Zhu D Wu BF Shi HB . Transarterial chemoembolization combined with lenvatinib and sintilimab vs lenvatinib alone in intermediate-advanced hepatocellular carcinoma. World J Gastrointest Oncol. (2025) 17:96267. doi: 10.4251/wjgo.v17.i1.96267, PMID: 39817120 PMC11664616

[52] Liu J Wang P Shang L Zhang Z Tian Y Chen X . TACE plus tyrosine kinase inhibitors and immune checkpoint inhibitors versus TACE plus tyrosine kinase inhibitors for the treatment of patients with hepatocellular carcinoma: a meta-analysis and trial sequential analysis. Hepatol Int. (2024) 18:595–609. doi: 10.1007/s12072-023-10591-0, PMID: 37843788

[53] Chen QF Chen S Zhao M . The synergistic mechanisms and prospects of transarterial chemoembolization combined with immunotherapy for hepatocellular carcinoma. J Hepatocell Carcinoma. (2025) 12:841–54. doi: 10.2147/JHC.S514881, PMID: 40322278 PMC12050036

[54] Encarnação CC Faria GM Franco VA Botelho LGX Moraes JA Renovato-Martins M . Interconnections within the tumor microenvironment: extracellular vesicles as critical players of metabolic reprogramming in tumor cells. J Cancer Metastasis Treat. (2024) 10:28. doi: 10.20517/2394-4722.2024.78

[55] Chen G Huang AC Zhang W Zhang G Wu M Xu W . Exosomal PD-L1 contributes to immunosuppression and is associated with anti-PD-1 response. Nature. (2018) 560:382–6. doi: 10.1038/s41586-018-0392-8, PMID: 30089911 PMC6095740

[56] Matsuki M Hoshi T Yamamoto Y Ikemori-Kawada M Minoshima Y Funahashi Y . Lenvatinib inhibits angiogenesis and tumor fibroblast growth factor signaling pathways in human hepatocellular carcinoma models. Cancer Med. (2018) 7:2641–53. doi: 10.1002/cam4.1517, PMID: 29733511 PMC6010799

[57] Huynh H Lee LY Goh KY Ong R Hao HX Huang A . Infigratinib mediates vascular normalization, impairs metastasis, and improves chemotherapy in hepatocellular carcinoma. Hepatology. (2019) 69:943–58. doi: 10.1002/hep.30481, PMID: 30575985 PMC6635738

[58] Zhu HD Li HL Huang MS Yang WZ Yin GW Zhong BY . Transarterial chemoembolization with PD-(L)1 inhibitors plus molecular targeted therapies for hepatocellular carcinoma (CHANCE001). Signal Transduct Target Ther. (2023) 8:58. doi: 10.1038/s41392-022-01235-0, PMID: 36750721 PMC9905571

[59] Sonkin D Thomas A Teicher BA . Cancer treatments: Past, present, and future. Cancer Genet. (2024) 286-287:18–24. doi: 10.1016/j.cancergen.2024.06.002, PMID: 38909530 PMC11338712

