# Supplementary material for: Efficacy of TACE plus tyrosine kinase inhibitors and immune checkpoint inhibitors in patients with unresectable hepatocellular carcinoma: a systematic review and meta-analysis
Source: Front Oncol. 2025 Dec 11;15:1707622. doi: 10.3389/fonc.2025.1707622 (PMC12739551; doi:10.3389/fonc.2025.1707622)
Supplement: Supplementary file 1 [file DataSheet1.pdf]

Supplementary Material

1 Supplementary Draft of search strategy

1.1 Search strategy in Pubmed

(((("Liver Neoplasms"[Mesh]) OR (((((((((((((((((((((Hepatic Neoplasms[Title/Abstract]) OR (Hepatic Neoplasm[Title/Abstract])) OR (Neoplasm, Hepatic[Title/Abstract])) OR (Neoplasms, Hepatic[Title/Abstract])) OR (Neoplasms, Liver[Title/Abstract])) OR (Liver Neoplasm [Title/Abstract])) OR (Neoplasm, Liver[Title/Abstract])) OR (Cancer of Liver[Title/Abstract])) OR (Liver Cancer[Title/Abstract])) OR (Cancer, Liver[Title/Abstract])) OR (Cancers, Liver [Title/Abstract])) OR (Liver Cancers[Title/Abstract])) OR (Hepatocellular Cancer [Title/Abstract])) OR (Cancers, Hepatocellular[Title/Abstract])) OR (Hepatocellular Cancers[Title/Abstract])) OR (Cancer of the Liver[Title/Abstract])) OR (Cancer, Hepatocellular [Title/Abstract])) OR (Hepatic Cancer[Title/Abstract])) OR (Cancer, Hepatic[Title/Abstract])) OR (Cancers, Hepatic [Title/ Abstract])) OR (Hepatic Cancers[Title/Abstract])) OR (HCC[Title/Abstract]))) AND (("Chemoembolization, Therapeutic"[Mesh]) OR ((((Therapeutic Chemoembolization[Title/Abstract]) OR (Chemoembolizations,Therapeutic[Title/Abstract]))OR(Therapeutic Chemoembolizations [Title/Abstract])) OR (TACE[Title/Abstract])))) AND (("Tyrosine Kinase Inhibitors"[Mesh]) OR (((((((((((Inhibitors, Tyrosine Kinase[Title/Abstract]) OR (Kinase Inhibitors, Tyrosine [Title/Abstract])) OR (Tyrosine Protein Kinase Inhibitors[Title/Abstract])) OR (Tyrosine Kinase Inhibitor[Title/Abstract])) OR (TKI Tyrosine Kinase Inhibitors[Title/Abstract])) OR (Sorafenib [Title/Abstract])) OR (Len- vatinib[Title/Abstract])) OR (Regorafenib[Title/Abstract])) OR (Apatinib[Title/Abstract])) OR (Donafenib[Title/Abstract])) OR (Cabozantinib[Title/Abstract])))) AND (("Immune Checkpoint Inhibitors"[Mesh]) OR ((((((((((((((((((((((((((((((((((Checkpoint Inhibitors, Immune[Title/Abstract]) OR (Immune Checkpoint Blockers[Title/Abstract])) OR (Checkpoint Blockers, Immune[Title/Abstract])) OR (Immune Checkpoint Inhibitor[Title/Abstract])) OR (Checkpoint Inhibitor, Immune[Title/Abstract])) OR (CTLA-4 Inhibitors [Title/Abstract])) OR (CTLA 4 Inhibitors[Title/Abstract])) OR (Cytotoxic T-Lymphocyte-Associated Protein 4 Inhibitors [Title/Abstract])) OR (Cytotoxic T Lymphocyte Associated Protein 4 Inhibitors [Title/Abstract])) OR (Cytotoxic T-Lymphocyte-Associated Protein 4 Inhibitor[Title/Abstract])) OR (Cytotoxic T Lymphocyte Associated Protein 4 Inhibitor[Title/Abstract])) OR (CTLA-4 Inhibitor[Title/Abstract])) OR (CTLA 4 Inhibitor[Title/Abstract])) OR (PD-1 Inhibitors [Title/Abstract])) OR (PD 1 Inhibitors[Title/Abstract])) OR (Programmed Cell Death Protein 1 Inhibitor[Title/Abstract])) OR (Programmed Cell Death Protein 1 Inhibitors[Title/Abstract])) OR (PD-1 Inhibitor[Title/Abstract])) OR (Inhibitor, PD-1[Title/Abstract])) OR (PD 1 Inhibitor [Title/ Abstract])) OR (Immune Checkpoint Blockade[Title/Abstract])) OR (Checkpoint Blockade, Immune[Title/Abstract])) OR (Immune Checkpoint Inhibition[Title/Abstract])) OR (Checkpoint Inhibition, Immune [Title/Abstract])) OR (PD-L1 Inhibitors[Title/Abstract])) OR (PD L1 Inhibitors [Title/Abstract])) OR (Programmed Death-Ligand 1 Inhibitors[Title/Abstract])) OR (Programmed Death Ligand 1 Inhibitors[Title/Abstract])) OR (PD-L1 Inhibitor[Title/Abstract])) OR (PD L1 Inhibitor [Title/Abstract])) OR (PD-1-PD-L1 Blockade[Title/Abstract])) OR (Blockade, PD-1-PD-L1 [Title/Abstract])) OR (PD 1 PD L1 Blockade[Title/Abstract])))

1.2 Search strategy in Cochrane library

#1 MeSH descriptor: [Liver Neoplasms] explode all trees

#2 (Liver Neoplasms):ti,ab,kw OR (Hepatic Neoplasms):ti,ab,kw OR (Hepatic Neoplasm):ti,ab,kw OR

(Neoplasm, Hepatic):ti,ab,kw OR (Neoplasms, Hepatic):ti,ab,kw OR (Neoplasms, Liver):ti,ab,kw OR (Liver Neoplasm):ti,ab,kw OR (Neoplasm, Liver):ti,ab,kw OR (Cancer of Liver):ti,ab,kw OR (Liver Cancer):ti,ab,kw OR (Cancer, Liver):ti,ab,kw OR (Cancers, Liver):ti,ab,kw OR (Liver Cancers):ti,ab,kw OR (Hepatocellular Cancer):ti,ab,kw OR (Cancers, Hepatocellular):ti,ab,kw OR (Hepatocellular Cancers):ti,ab,kw OR (Cancer of the Liver):ti,ab,kw OR (Cancer, Hepatocellular):ti,ab,kw OR (Hepatic Cancer):ti,ab,kw OR (Cancer, Hepatic):ti,ab,kw OR (Cancers, Hepatic):ti,ab,kw OR (Hepatic Cancers):ti,ab,kw OR (HCC):ti,ab,kw

#3 #1 OR #2

#4 MeSH descriptor: [Chemoembolization, Therapeutic] explode all trees

#5 (Therapeutic Chemoembolization):ti,ab,kw OR (Chemoembolizations, Therapeutic):ti,ab,kw OR (Therapeutic Chemoembolizations):ti,ab,kw OR (TACE):ti,ab,kw OR (Chemoembolization):ti,ab,kw

#6 #4 OR #5

#7 MeSH descriptor: [Tyrosine Kinase Inhibitors] explode all trees

#8 (Inhibitors, Tyrosine Kinase):ti,ab,kw OR (Kinase Inhibitors, Tyrosine):ti,ab,kw OR (Tyrosine Protein Kinase Inhibitors):ti,ab,kw OR (Tyrosine Kinase Inhibitor):ti,ab,kw OR (TKI Tyrosine Kinase Inhibitors):ti,ab,kw OR (TKI):ti,ab,kw

#9 #7 OR #8

#10 MeSH descriptor: [Immune Checkpoint Inhibitors] explode all trees

#11 (Cytotoxic T-Lymphocyte-Associated Protein 4 Inhibitor):ti,ab,kw OR (Cytotoxic T Lymphocyte Associated Protein 4 Inhibitors):ti,ab,kw OR (Cytotoxic T-Lymphocyte-Associated Protein 4 Inhibitors):ti,ab,kw OR (Cytotoxic T Lymphocyte Associated Protein 4 Inhibitor):ti,ab,kw OR (Programmed Cell Death Protein 1 Inhibitors):ti,ab,kw (PD 1 Inhibitors):ti,ab,kw OR (Programmed Cell Death Protein 1 Inhibitor):ti,ab,kw OR (PD 1 Inhibitor):ti,ab,kw OR (Checkpoint Inhibitors, Immune):ti,ab,kw OR (Immune Checkpoint Inhibitor):ti,ab,kw OR (Checkpoint Inhibitor, Immune):ti,ab,kw OR (Immune Checkpoint Blockers):ti,ab,kw OR (Checkpoint Blockers, Immune):ti,ab,kw OR (PD 1 PD L 1 Blockade):ti,ab,kw OR (Immune Checkpoint Blockade):ti,ab,kw OR (Checkpoint Blockade, Immune):ti,ab,kw OR (Immune Checkpoint Inhibition):ti,ab,kw OR (Checkpoint Inhibition, Immune):ti,ab,kw OR (Programmed Death-Ligand 1 Inhibitors):ti,ab,kw OR (Programmed Death Ligand 1 Inhibitors):ti,ab,kw OR (CTLA 4 Inhibitors):ti,ab,kw OR (CTLA-4 Inhibitors):ti,ab,kw OR (CTLA 4 Inhibitor):ti,ab,kw OR (CTLA-4):ti,ab,kw OR (CTLA 4):ti,ab,kw OR (PD-1):ti,ab,kw OR (PD 1):ti,ab,kw OR (PD-L1):ti,ab,kw OR (PD L1):ti,ab,kw OR (ICI):ti,ab,kw

#12 #10 OR #11

#13 #3 AND #6 AND #9 AND #12

2 Supplementary Figures

2.1 Supplement figure 1.

Summary sensitivity analysis plots for TACE + TKIs + ICIs vs TACE + TKIs (left); TACE + TKIs + ICIs vs TACE (right).

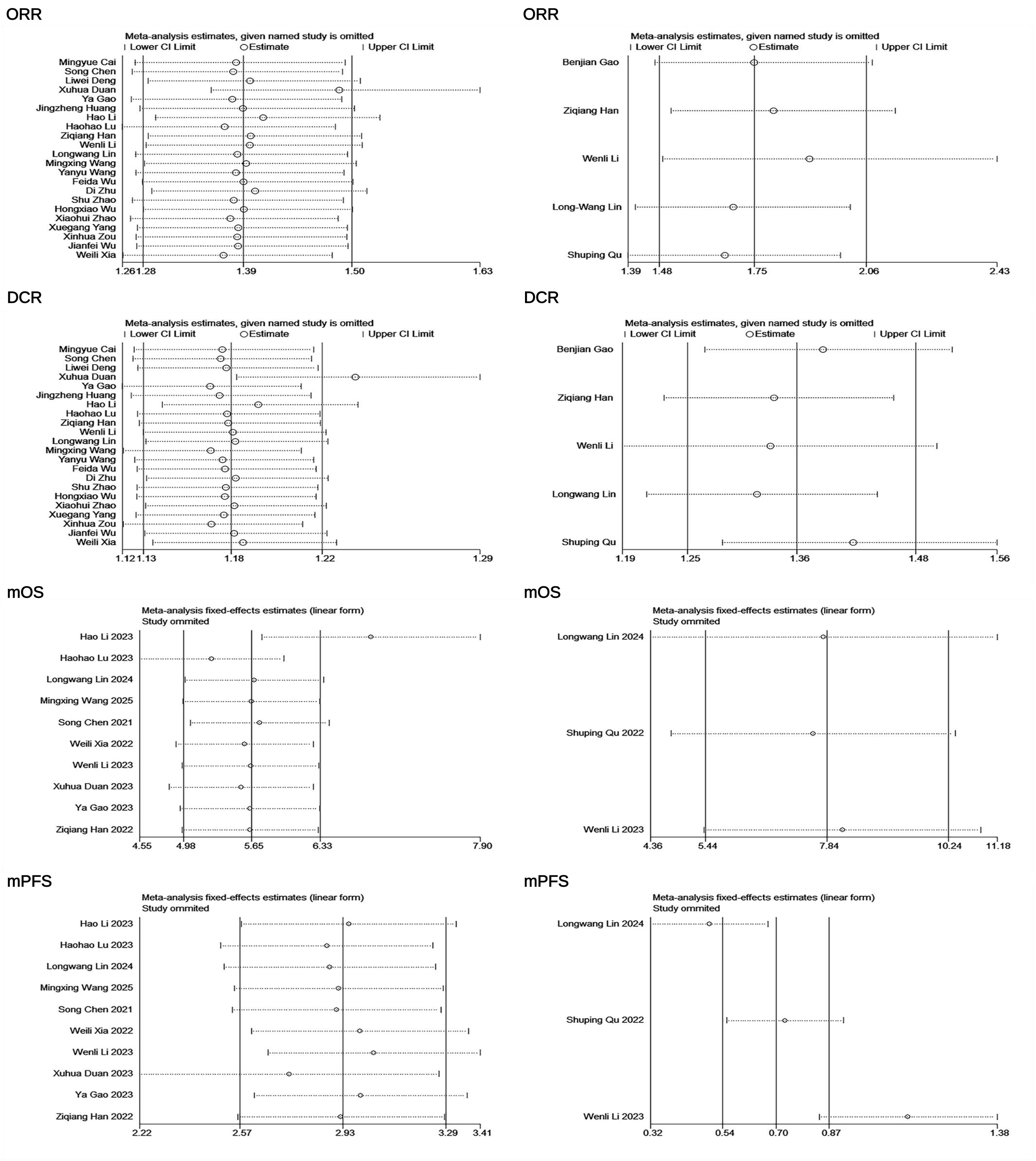
ORR
Meta-analysis estimates, given named study is omitted
Lower CI Limit
Estimate
Upper CI Limit
Mingyue Cai
Song Chen
Liwei Deng
Xuhua Duan
Ya Gao
Jingzheng Huang
Hao Li
Haohao Lu
Ziqiang Han
Wenli Li
Longwang Lin
Mingxing Wang
Yanyu Wang
Feida Wu
Di Zhu
Shu Zhao
Hongxiao Wu
Xiaohui Zhao
Xuegang Yang
Xinhua Zou
Jianfei Wu
Weili Xia
1.26
1.28
1.39
1.50
1.63
ORR
Meta-analysis estimates, given named study is omitted
Lower CI Limit
Estimate
Upper CI Limit
Benjian Gao
Ziqiang Han
Wenli Li
Long-Wang Lin
Shuping Qu
1.39
1.48
1.75
2.06
2.43
DCR
Meta-analysis estimates, given named study is omitted
Lower CI Limit
Estimate
Upper CI Limit
Mingyue Cai
Song Chen
Liwei Deng
Xuhua Duan
Ya Gao
Jingzheng Huang
Hao Li
Haohao Lu
Ziqiang Han
Wenli Li
Longwang Lin
Mingxing Wang
Yanyu Wang
Feida Wu
Di Zhu
Shu Zhao
Hongxiao Wu
Xiaohui Zhao
Xuegang Yang
Xinhua Zou
Jianfei Wu
Weili Xia
1.12
1.13
1.18
1.22
1.29
DCR
Meta-analysis estimates, given named study is omitted
Lower CI Limit
Estimate
Upper CI Limit
Benjian Gao
Ziqiang Han
Wenli Li
Longwang Lin
Shuping Qu
1.19
1.25
1.36
1.48
1.56
mOS
Meta-analysis fixed-effects estimates (linear form)
Study ommited
Hao Li 2023
Haohao Lu 2023
Longwang Lin 2024
Mingxing Wang 2025
Song Chen 2021
Weili Xia 2022
Wenli Li 2023
Xuhua Duan 2023
Ya Gao 2023
Ziqiang Han 2022
4.55
4.98
5.65
6.33
7.90
mOS
Meta-analysis fixed-effects estimates (linear form)
Study ommited
Longwang Lin 2024
Shuping Qu 2022
Wenli Li 2023
4.36
5.44
7.84
10.24
11.18
mPFS
Meta-analysis fixed-effects estimates (linear form)
Study ommited
Hao Li 2023
Haohao Lu 2023
Longwang Lin 2024
Mingxing Wang 2025
Song Chen 2021
Weili Xia 2022
Wenli Li 2023
Xuhua Duan 2023
Ya Gao 2023
Ziqiang Han 2022
2.22
2.57
2.93
3.29
3.41
mPFS
Meta-analysis fixed-effects estimates (linear form)
Study ommited
Longwang Lin 2024
Shuping Qu 2022
Wenli Li 2023
0.32
0.54
0.70
0.87
1.38

2.3 Supplementary Figure 2 .

Subgroup analysis of OS. Abbreviations: HBV, hepatitis B virus; AFP, alpha-fetoprotein; ECOG PS, Eastern Cooperative Oncology Group Performance Status; BCLC, Barcelona Clinic Liver Cancer; PVTT, portal vein tumor thrombosis; HVTT, Hepatic Vein Tumor Thrombus; EHM, Extrahepatic metastasis.

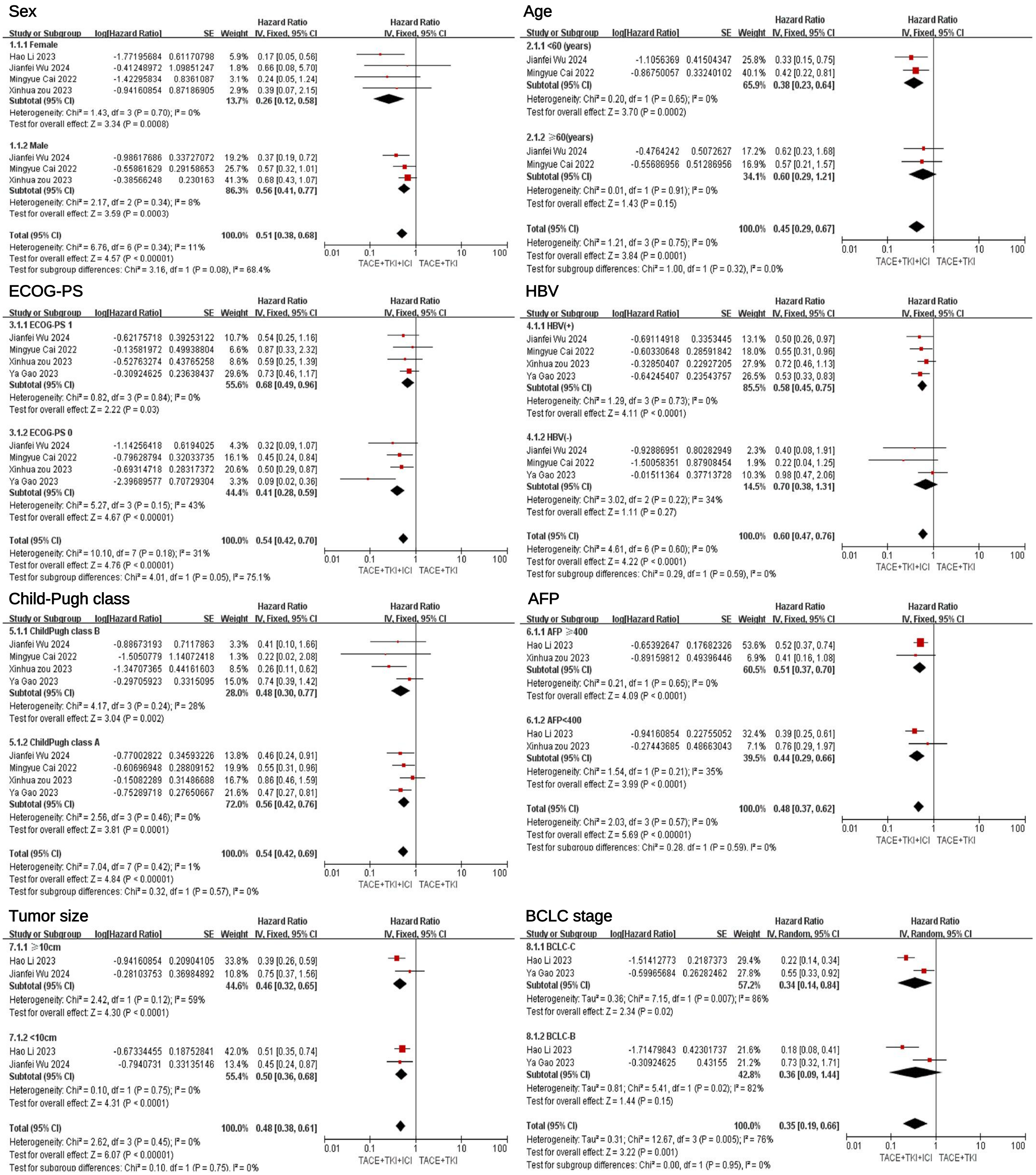

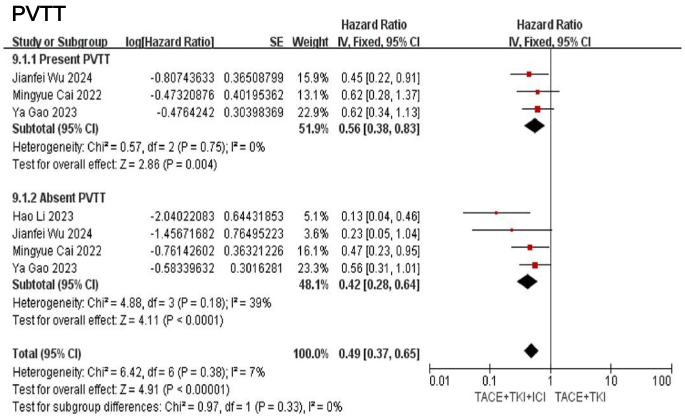

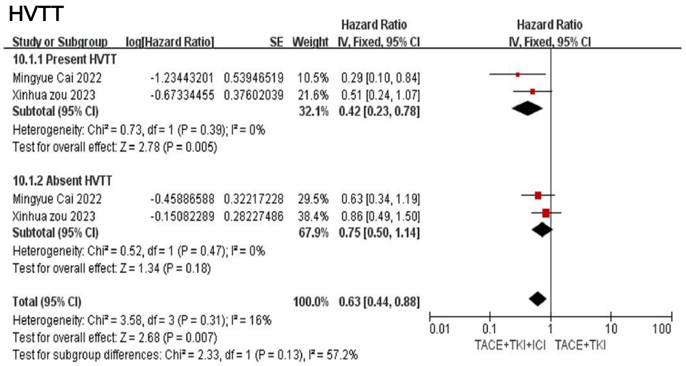

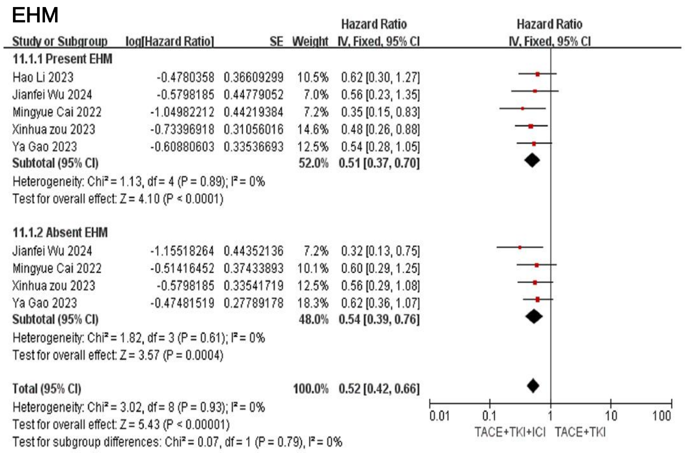

2.4 Supplementary Figure 3 .

Subgroup analysis of PFS. Abbreviations: HBV, hepatitis B virus; AFP, alpha-fetoprotein; ECOG PS, Eastern Cooperative Oncology Group Performance Status; BCLC, Barcelona Clinic Liver Cancer; PVTT, portal vein tumor thrombosis; HVTT, Hepatic Vein Tumor Thrombus; EHM, Extrahepatic metastasis.

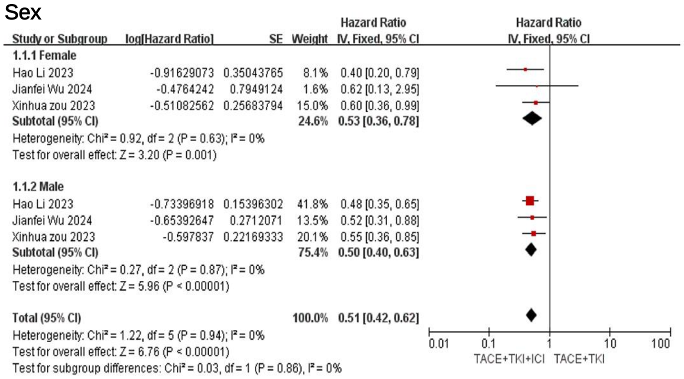

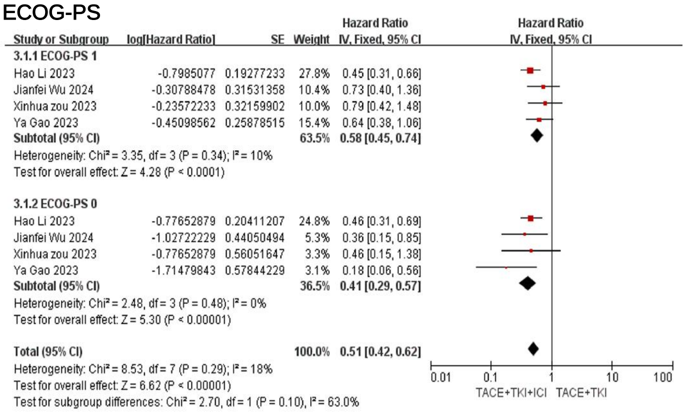

HBV

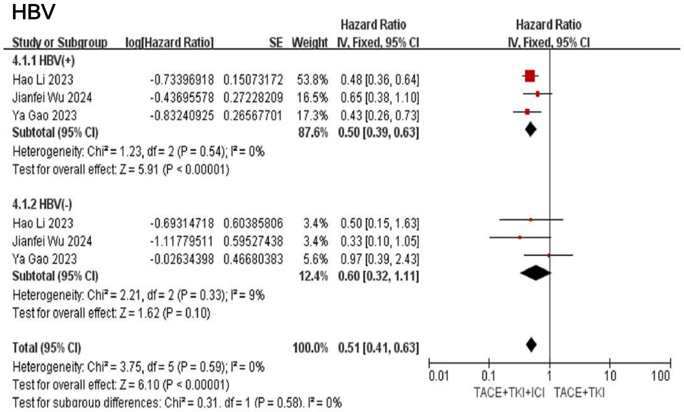

Child-Pugh class

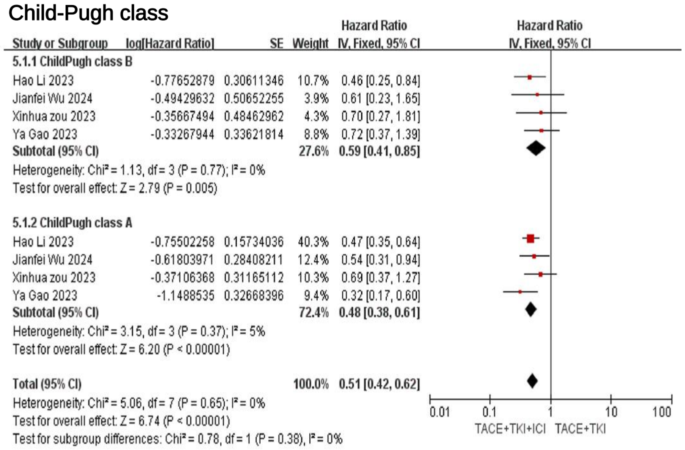

AFP

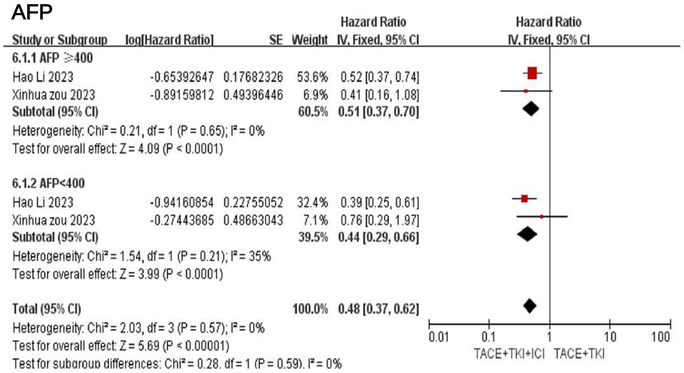

Tumor size

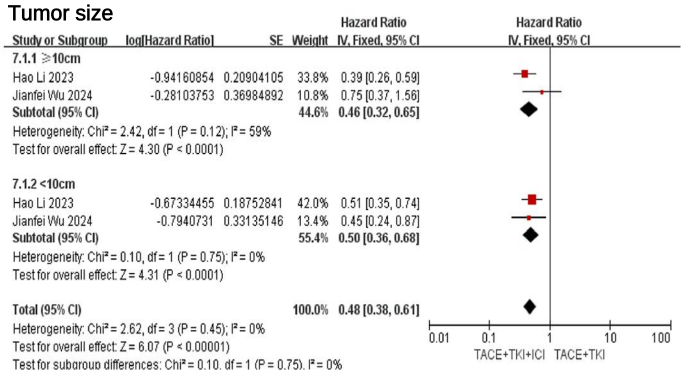

BCLC stage

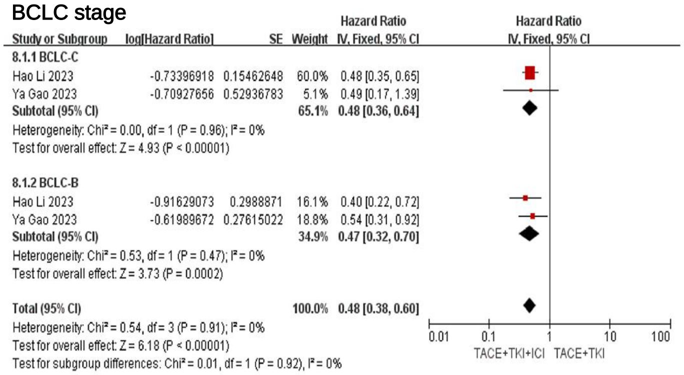

PVTT

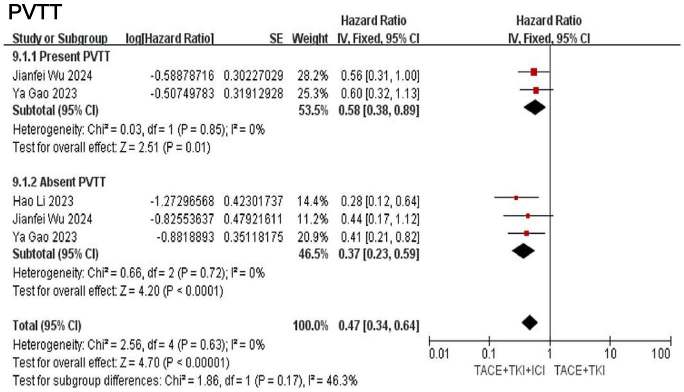

HVTT

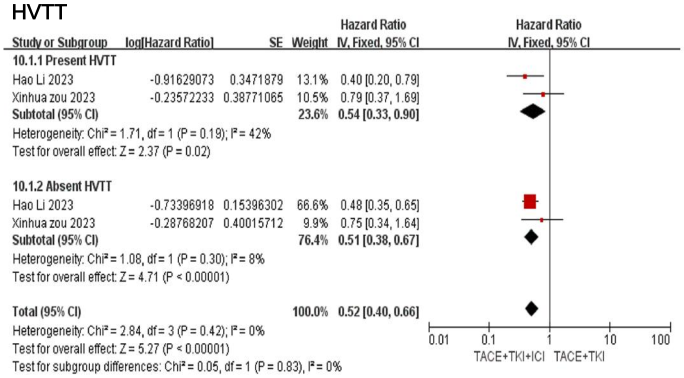

EHM

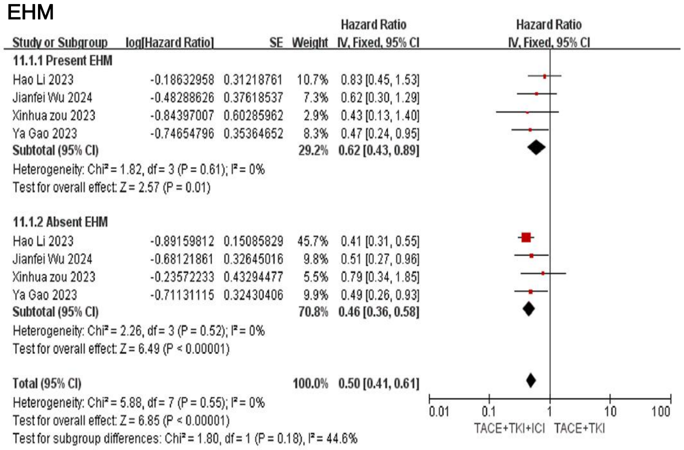

2.2 Supplementary Figure 4 .

Funnel plots of pooled analyses for TACE + TKIs + ICIs vs TACE + TKIs (left); TACE + TKIs + ICIs vs TACE (right).

ORR

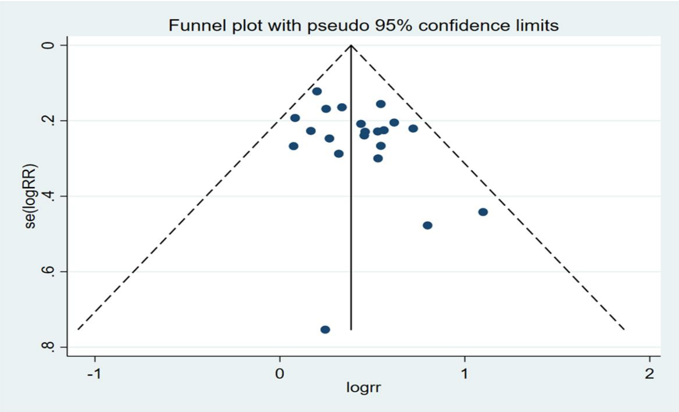

Begg's Test

```
adj. Kendall's Score (P-Q) =      28
        Std. Dev. of Score =   33.12
         Number of Studies =      21
                         z =    0.85
                  Pr > |z| =   0.398
                         z =    0.82 (continuity corrected)
                  Pr > |z| =   0.415 (continuity corrected)
```

Egger's test

Std_Eff	Coef.	Std. Err.	t	P>|t|	[95% Conf.	Interval]
slope	.1635937	.1312941	1.25	0.228	-.111208	.4383955
bias	1.080963	.6070848	1.78	0.091	-.1896803	2.351606

ORR

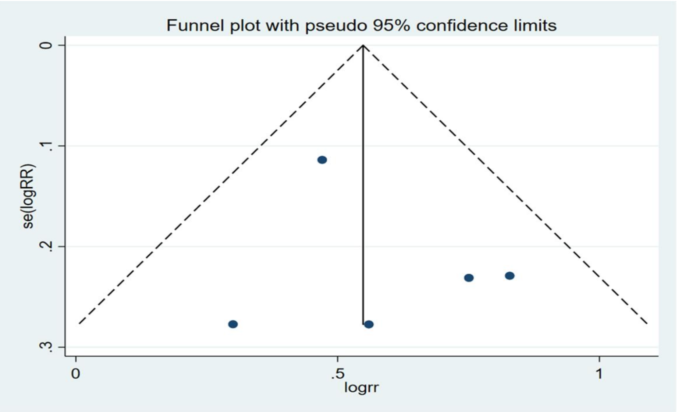

Begg's Test

```
adj. Kendall's Score (P-Q) =       0
        Std. Dev. of Score =    4.08
         Number of Studies =       5
                         z =    0.00
                  Pr > |z| =   1.000
                         z =   -0.24 (continuity corrected)
                  Pr > |z| =   1.000 (continuity corrected)
```

Egger's test

Std_Eff	Coef.	Std. Err.	t	P>|t|	[95% Conf.	Interval]
slope	.4050982	.232418	1.74	0.180	-.3345596	1.144756
bias	.819514	1.233995	0.66	0.554	-3.10761	4.746638

DCR

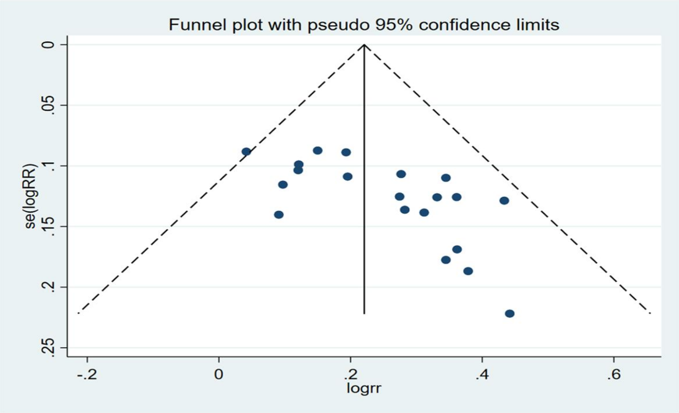

Begg's Test

```
adj. Kendall's Score (P-Q) =      82
        Std. Dev. of Score =   30.82
         Number of Studies =      20
                         z =    2.66
                  Pr > |z| =   0.008
                         z =    2.63 (continuity corrected)
                  Pr > |z| =   0.009 (continuity corrected)
```

Egger's test

Std_Eff	Coef.	Std. Err.	t	P>|t|	[95% Conf.	Interval]
slope	-.0918055	.0903079	-1.02	0.323	-.2815354	.0979244
bias	2.720442	.7650545	3.56	0.002	1.113122	4.327762

DCR

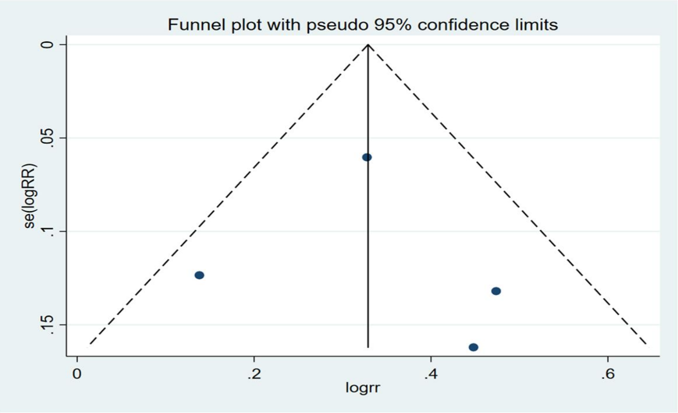

Begg's Test

```
adj. Kendall's Score (P-Q) =       2
        Std. Dev. of Score =    2.94
         Number of Studies =       4
                         z =    0.68
                  Pr > |z| =   0.497
                         z =    0.34 (continuity corrected)
                  Pr > |z| =   0.734 (continuity corrected)
```

Egger's test

Std_Eff	Coef.	Std. Err.	t	P>|t|	[95% Conf.	Interval]
slope	.2904023	.1733341	1.68	0.236	-.4553943	1.036199
bias	.4362471	1.809682	0.24	0.832	-7.350184	8.222678

mOS

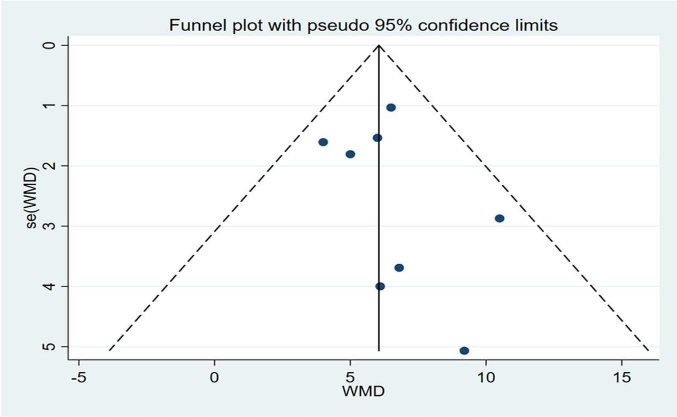

Begg's Test

```
adj. Kendall's Score (P-Q) =       6
        Std. Dev. of Score =    8.08
         Number of Studies =       8
                         z =    0.74
                  Pr > |z| =   0.458
                         z =    0.62 (continuity corrected)
                  Pr > |z| =   0.536 (continuity corrected)
```

Egger's test

Std_Eff	Coef.	Std. Err.	t	P>|t|	[95% Conf.	Interval]
slope	5.139082	1.23576	4.16	0.006	2.115287	8.162877
bias	.5564156	.6700743	0.83	0.438	-1.083197	2.196028

mOS

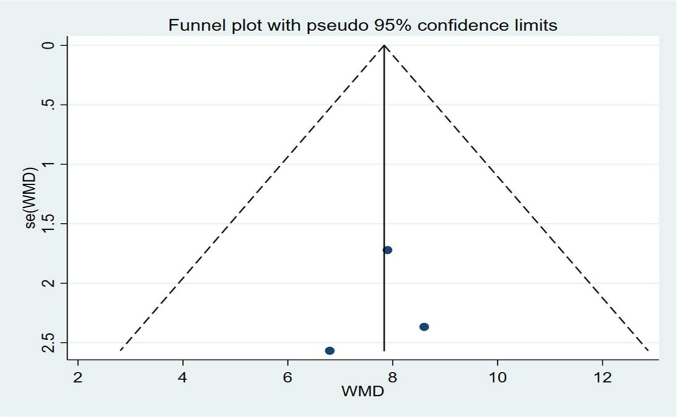

Begg's Test

```
adj. Kendall's Score (P-Q) =      -1
        Std. Dev. of Score =    1.91
         Number of Studies =       3
                         z =   -0.52
                  Pr > |z| =   0.602
                         z =    0.00 (continuity corrected)
                  Pr > |z| =   1.000 (continuity corrected)
```

Egger's test

Std_Eff	Coef.	Std. Err.	t	P>|t|	[95% Conf.	Interval]
slope	8.841081	3.431469	2.58	0.236	-34.75987	52.44203
bias	-.481155	1.618825	-0.30	0.816	-21.05027	20.08796

mPFS

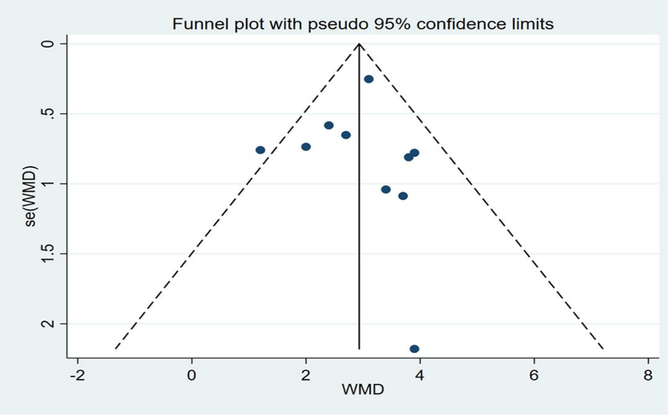

Begg's Test

```
adj. Kendall's Score (P-Q) =       3
        Std. Dev. of Score =   11.18
         Number of Studies =      10
                         z =    0.27
                  Pr > |z| =   0.788
                         z =    0.18 (continuity corrected)
                  Pr > |z| =   0.858 (continuity corrected)
```

Egger's test

Std_Eff	Coef.	Std. Err.	t	P>|t|	[95% Conf.	Interval]
slope	2.975974	.4293406	6.93	0.000	1.985913	3.966035
bias	-.0886234	.7405104	-0.12	0.908	-1.796243	1.618997

mPFS

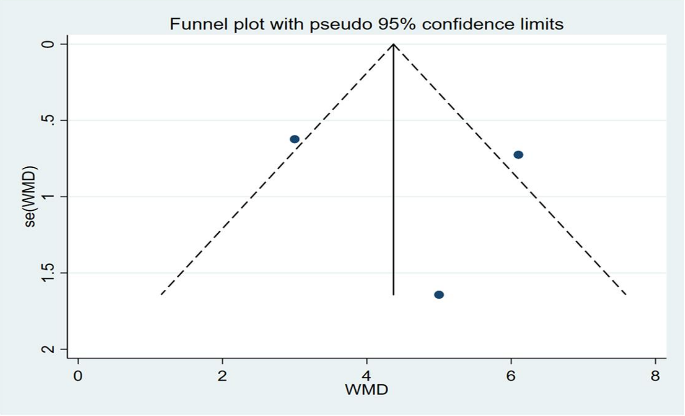

Begg's Test

```
adj. Kendall's Score (P-Q) =       1
        Std. Dev. of Score =    1.91
         Number of Studies =       3
                         z =    0.52
                  Pr > |z| =   0.602
                         z =    0.00 (continuity corrected)
                  Pr > |z| =   1.000 (continuity corrected)
```

Egger's test

Std_Eff	Coef.	Std. Err.	t	P>|t|	[95% Conf.	Interval]
slope	3.106833	4.21553	0.74	0.596	-50.45655	56.67022
bias	1.702552	5.358052	0.32	0.804	-66.37795	69.78306
